# Whole-exome sequencing association study reveals genetic effects on tumor microenvironment components in nasopharyngeal carcinoma

**DOI:** 10.1172/JCI182768

**Published:** 2025-01-02

**Authors:** Yanni Zeng, Chun-Ling Luo, Guo-Wang Lin, Fugui Li, Xiaomeng Bai, Josephine Mun-Yee Ko, Lei Xiong, Yang Liu, Shuai He, Jia-Xin Jiang, Wen-Xin Yan, Enya Hui Wen Ong, Zheng Li, Ya-Qing Zhou, Yun-He Zhou, An-Yi Xu, Shu-Qiang Liu, Yun-Miao Guo, Jie-Rong Chen, Xi-Xi Cheng, Yu-Lu Cao, Xia Yu, Biaohua Wu, Pan-Pan Wei, Zhao-Hui Ruan, Qiu-Yan Chen, Lin-Quan Tang, James D. McKay, Wei-Hua Jia, Hai-Qiang Mai, Soon Thye Lim, Jian-Jun Liu, Dong-Xin Lin, Chiea Chuen Khor, Melvin Lee Kiang Chua, Mingfang Ji, Maria Li Lung, Yi-Xin Zeng, Jin-Xin Bei

**Affiliations:** 1 State Key Laboratory of Oncology in South China, Guangdong Key Laboratory of Nasopharyngeal Carcinoma Diagnosis and Therapy, Guangdong Provincial Clinical Research Center for Cancer, Sun Yat-sen University Cancer Center - Zhongshan School of Medicine,; 2 Faculty of Forensic Medicine, Guangdong Province Translational Forensic Medicine Engineering Technology Research Center, and; 3 Guangdong Province Key Laboratory of Brain Function and Disease, Zhongshan School of Medicine, Sun Yat-sen University, Guangzhou, China.; 4 Department of Experimental Research, Sun Yat-sen University Cancer Center, Guangzhou, China.; 5 Department of Laboratory Medicine, Zhujiang Hospital, Southern Medical University, Guangzhou, Guangdong, China.; 6 Cancer Research Institute of Zhongshan City, Zhongshan City People’s Hospital, Zhongshan, China.; 7 Department of Clinical Oncology, School of Clinical Medicine, University of Hong Kong, Hong Kong SAR, China.; 8 Precision Radiotherapeutics Oncology Programme, Division of Medical Sciences, National Cancer Centre Singapore, Singapore.; 9 Genome Institute of Singapore (GIS), Agency for Science, Technology and Research (A*STAR), Singapore.; 10 Zhanjiang Institute of Clinical Medicine, Central People’s Hospital of Zhanjiang, Guangdong Medical University Zhanjiang Central Hospital, Zhanjiang, China.; 11 Department of Laboratory Medicine, Guangdong Provincial People’s Hospital (Guangdong Academy of Medical Sciences), Southern Medical University, Guangzhou, China.; 12 Genomic Epidemiology Branch, International Agency for Research on Cancer/World Health Organization (IARC/WHO), Lyon, France.; 13 Department of Medical Oncology, National Cancer Centre Singapore, Singapore.; 14 Laboratory of Human Genomics, Genome Institute of Singapore, Agency for Science, Technology and Research (A*STAR), Singapore.; 15 Ophthalmology & Visual Sciences Academic Clinical Programme, Duke-National University of Singapore Medical School, Singapore.; 16 Singapore Eye Research Institute, Discovery Tower, Level 6, The Academia, Singapore.; 17 Department of Head and Neck and Thoracic Radiation Oncology, National Cancer Centre Singapore, Singapore.; 18 Oncology Academic Clinical Programme, Duke-NUS Medical School, Singapore.; 19 Sun Yat-sen University Institute of Advanced Studies Hong Kong, Science Park, Hong Kong SAR, China.

**Keywords:** Genetics, Oncology, Cancer, Genetic variation

## Abstract

Nasopharyngeal carcinoma (NPC) presents a substantial clinical challenge due to the limited understanding of its genetic underpinnings. Here we conduct the largest scale whole-exome sequencing association study of NPC to date, encompassing 6,969 NPC cases and 7,100 controls. We unveil 3 germline genetic variants linked to NPC susceptibility: a common rs2276868 in *RPL14*, a rare rs5361 in *SELE*, and a common rs1050462 in *HLA-B*. We also underscore the critical impact of rare genetic variants on NPC heritability and introduce a refined composite polygenic risk score (rcPRS), which outperforms existing models in predicting NPC risk. Importantly, we reveal that the polygenic risk for NPC is mediated by EBV infection status. Utilizing a comprehensive multiomics approach that integrates both bulk-transcriptomic (*n* = 356) and single-cell RNA sequencing (*n* = 56) data with experimental validations, we demonstrate that the *RPL14* variant modulates the EBV life cycle and NPC pathogenesis. Furthermore, our data indicate that the *SELE* variant contributes to modifying endothelial cell function, thereby facilitating NPC progression. Collectively, our study provides crucial insights into the intricate genetic architecture of NPC, spotlighting the vital interplay between genetic variations and tumor microenvironment components, including EBV and endothelial cells, in predisposing to NPC. This study opens new avenues for advancements in personalized risk assessments, early diagnosis, and targeted therapies for NPC.

## Abstract 2

Extensive DNA analysis links genetic factors to virus and specialized component in tumor microenvironment in Nasopharyngeal carcinoma

## Introduction

Nasopharyngeal carcinoma (NPC) is a highly lethal malignancy predominantly affecting East and Southeast Asia (1), where it accounts for approximately 70% of new cases worldwide annually (1), with the highest incidence rate (9.69/100,000) observed in southern China (2). Familial clustering in a notable proportion (approximately 3.64%–19%) of NPC patients from diverse populations suggests a marked hereditary component to NPC risk (3). Genetic susceptibility, EBV infection, and environmental exposures are believed to play essential roles in NPC development (1).

Despite efforts through linkage analyses, regional and genome-wide association studies (GWAS) that have identified risk variants (4–9), particularly in the HLA region, a substantial portion (>90%) of NPC heritability (approximately 10%–61.3%) remains unexplained (9, 10). This “missing heritability” may partially result from insufficient statistical power or coverage of risk variants with lower to rare frequencies, of which the detection requires whole-genome sequencing (WGS) or whole-exome sequencing (WES) (11, 12). By far, only a few WES studies have explored the contribution of rare variants, including those residing in *MST1R*, *RAD54L*, and *POLN*, to NPC risk (13–15). However, these findings either did not reach exome-wide statistical significance or have not been independently validated, likely due to limited sample sizes or genetic heterogeneity across the population. Therefore, the genetic architecture involving both common and rare variants in NPC risk remains unclear.

The critical role of EBV molecules in the screening and diagnosis of NPC is well documented, with the virus infecting epithelial cells and playing a crucial role in NPC progression (16, 17). Malignant epithelial cells with EBV infection and various stromal and immune cell types jointly foster a complex tumor microenvironment (TME), which results in notable intra- and intertumor heterogeneity in NPC (18, 19). Despite these insights, the influence of genetic variations on EBV and other TME components and how this contributes to the missing heritability of NPC remain elusive.

This study aims to bridge the gap in our comprehension of NPC’s genetic architecture and to translate these genetic insights to better understand its pathological mechanisms and risk prediction. We first conducted a 2-stage association study on 14,069 individuals of southern Chinese descent from Guangdong (GD-SYSUCC and GD-ZS), Hong Kong (HK), and Singapore, representing the most extensive WES and capture-sequencing (Cap-seq) analyses on NPC to date. Furthermore, our study proposes an improved strategy for NPC risk prediction, utilizing WES-derived genetic variants and EBV infection status. Moreover, by integrating both bulk transcriptomic and single-cell RNA-Seq (scRNA-Seq) analyses with experimental validations, our study unveils the critical contributions of both common and rare genetic variants to NPC heritability and sheds light on how these variants functionally influence EBV-related pathogenesis and TME components, thereby leading to varied NPC susceptibilities.

## Results

### Association of single variants with NPC susceptibility.

**Figure 1:**
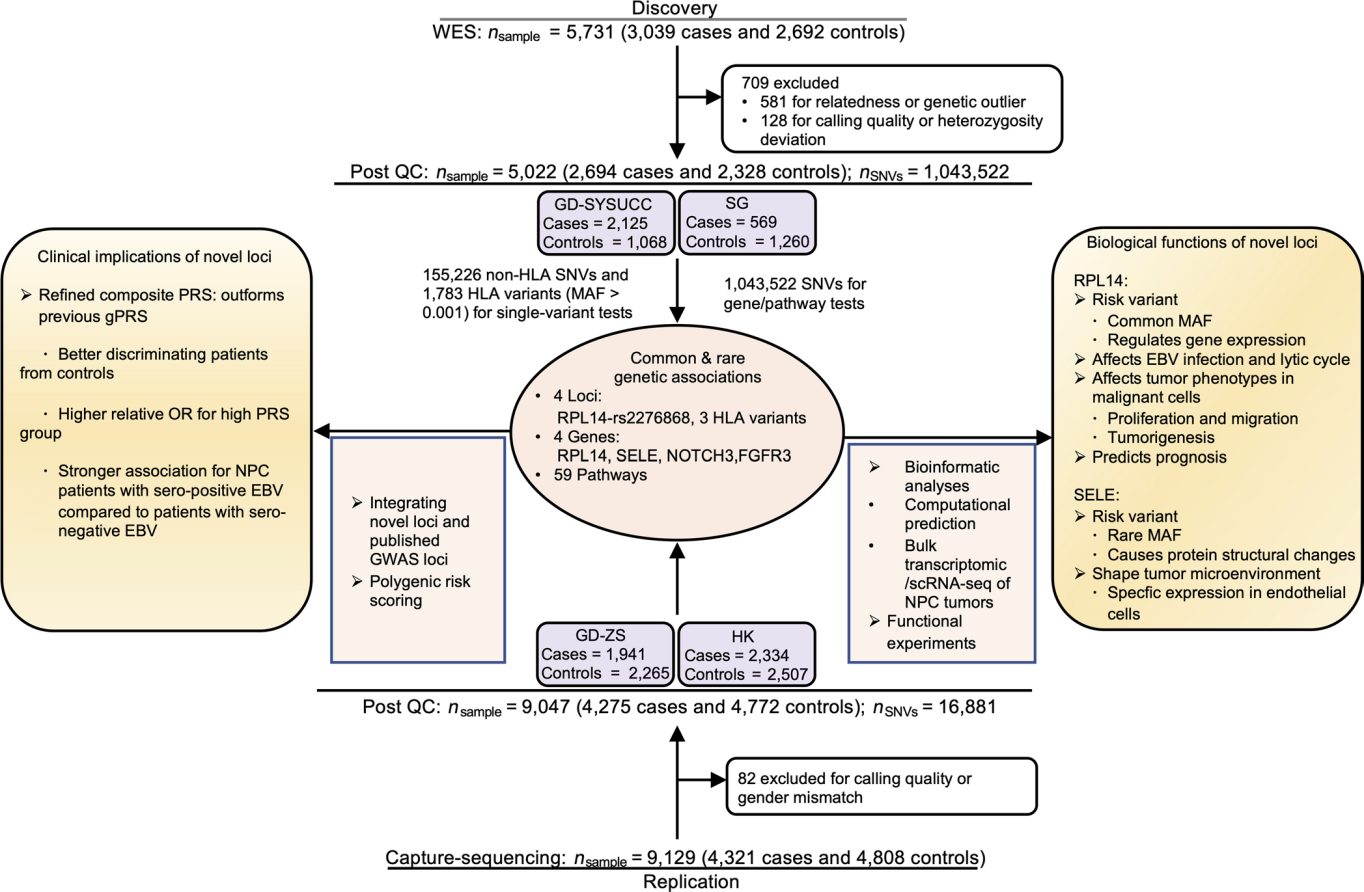
Study overview. A 2-stage association study design was applied to investigate the genetic factors associated with NPC. In the discovery stage, a total of 5,022 samples, including the GD-SYSUCC cohort from Guangdong in China and the SG cohort from Singapore, were genotyped using WES and analyzed to identify independent variants, genes, and pathways associated with NPC. The associations were subsequently validated in the replication stage, which included 9,047 samples from the GD-ZS cohort from Guangdong and the HK cohort from Hong Kong. Bioinformatic analyses and functional experiments were conducted to explore the clinical application and the biological functions of the identified loci.

To identify susceptibility loci for NPC at the single variant level, we conducted a classical case-control association study using a 2-stage design. In the discovery stage, we first obtained genotype data through WES of 2,694 NPC cases (ALLNPC, Supplemental Table 1) and 2,328 healthy controls of Han Chinese descent from southern China (GD-SYSUCC) and Singapore (SG) (see Methods and Figure 1). Importantly, we included a subset of 409 cases with a familial NPC history (FHNPC) to enhance the likelihood of identifying genetic loci (20, 21). After applying stringent quality controls, we identified 1,043,522 single nucleotide variants (SNVs), including 362,993 potentially functional or pathogenic variants and 335,163 variants that were not present in major genome databases (22, 23).

Subsequently, we performed a single-variant exome-wide association study (EWAS), adjusting for the top 5 principal components (PCs) and sex, on 155,226 non-HLA SNVs and 1,783 HLA variants with a minor allele frequency (MAF) of at least 0.001 in both the ALLNPC cases and FHNPC cases compared with controls (see Methods; Supplemental Figure 1A and Supplemental Figure 2; supplemental material available online with this article; https://doi.org/10.1172/JCI182768DS1). The EWAS identified 242 variants associated with NPC risk in the ALLNPC group and 128 in the FHNPC group, each surpassing the significance threshold after Bonferroni’s correction for exome-wide level tests (*P_Bonferroni_threshold_* < 3.2 × 10^–7^, *n_tested_variant_* = 157,009; Supplemental Figure 1B). Notably, we discovered a non-HLA locus with several significantly associated SNVs (P < 3.2 × 10^–7^). Among these, the sentinel SNV rs2276868, located in the 5′-untranslated region (5′-UTR) of the *RPL14* gene, exhibited the strongest associations in both the FHNPC (odds ratio [OR] _FHNPC_ =1.575, *P_FHNPC_* = 2.3 × 10^–8^) and the ALLNPC groups (OR_ALLNPC_ = 1.242, *P_ALLNPC_* = 4.2 × 10^–7^; Table 1), indicating a particularly pronounced genetic effect in familial cases. Further conditional analysis pinpointed that rs2276868 explained all the observed associations at the *RPL14* locus, as these associations vanished upon adjusting for rs2276868 (Supplemental Tables 2 and 3). Additionally, we identified significant associations with HLA variants, among which stepwise conditional analysis uncovered 3 independent associations, including rs1050462 in *HLA-B* and 2 known associations with *HLA-A: A_62_Q* and HLA-DQB1*03:01:01 (9, 24) (Table 1 and Supplemental Tables 2 and 4). Additionally, sensitive analyses incorporating age as a covariate for available samples demonstrated that our findings remain robust (Supplemental Table 5).

In the replication stage, we genotyped 2 independent Southern Chinese cohorts consisting of 9,047 samples from HK and GD-ZS, using Cap-seq to target the relevant SNVs and exon regions (see Methods). Logistic regression analysis confirmed significant associations of the 4 variants in *RPL14* and *HLA* loci in these 2 cohorts as well as a subset of 604 patients with family history in HK cohort (*P_Bonferroni_threshold_* < 0.0125, *n_tested_variant_* = 4; Table 1), indicating successful replication of these findings in both loci. Metaanalysis combining the discovery and replication samples of 6,969 cases and 7,100 controls further corroborated these variants as significantly associated with NPC risk at genome-wide significance level (*P* < 5 × 10^–8^; Table 1).

### Association of genes and pathways with NPC susceptibility.

To further explore the genetic structure underlying NPC susceptibility, we assessed the cumulative genetic effects of both rare and common variants within individual genes on NPC susceptibility by conducting a gene-based association analysis using the discovery dataset. This analysis employed an ensemble of 4 algorithms that consider all SNVs within genic regions or only coding-affecting SNVs for each gene (see Methods). This comprehensive approach pinpointed a significant association between *RPL14* and NPC risk, surpassing the stringent significance threshold (*P_Bonferroni_threshold_* < 2.3 × 10^–6^, *n_tested_genes_* = 22,228; Table 2). Additionally, this approach successfully validated previously reported associations with *POLN*, *RAD54L*, *EML2*, and *MST1R* (*P_FDR_threshold_* < 0.026, *n_tested_gene_* = 6; Supplemental Table 6).

**Figure 2:**
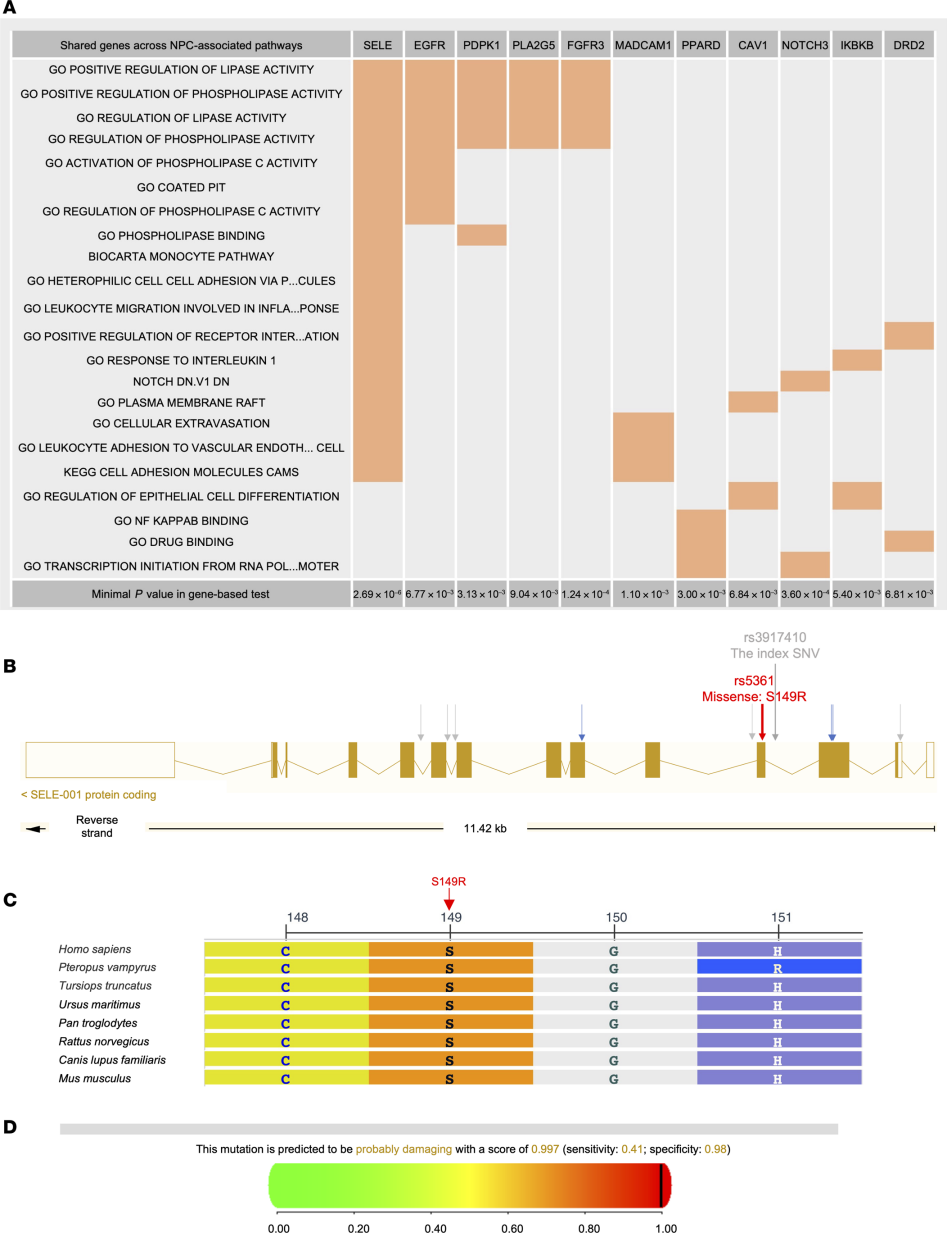
The sentinel genes from significant pathways associated with NPC and functional implication of *SELE* variants. (**A**). Sentinel genes for NPC-associated pathways. Genes indicated on top are highlighted in orange if they are part of a specific pathway. The listed genes are those belonging to at least 2 NPC-associated pathways and exhibiting a gene-based association *P* value below 0.01. (**B**) Locations of the rare variants associated with NPC at significance level of *P* < 1 × 10^–4^ within the genic regions of *SELE*. Gray arrows denote noncoding variants, blue arrows represent synonymous coding variants, and the red arrow indicates nonsynonymous coding variant predicted as “deleterious”. The rs5361 minor allele introduces a missense mutation at position 149, resulting in an aa substitution from S to R. The index SNV rs3917410 showed the most significant *P* value in the association tests in the discovery stage. (**C**) Comparative analysis of aa sequences across multiple species at position 149 and adjacent regions in SELE using the NCBI Multiple Sequence Alignment Viewer. (**D**) PolyPhen-2 prediction of SELE-S149R mutation. The variant is predicted as “probably damaging” with a score of 0.997, indicating a high likelihood of functional impact (sensitivity: 0.41; specificity: 0.98). The score ranges from 0 (benign) to 1 (damaging).

We also performed a pathway-based analysis to examine the cumulative genetic effects of 6,204 curated molecular pathways on NPC susceptibility using the discovery dataset (see Methods). This analysis identified a total of 59 pathways significantly associated with NPC in either the ALLNPC or FHNPC groups, adhering to the FDR threshold (*P_FDR_threshold_* < 3.09 × 10^–4^; Supplemental Figure 3 and Supplemental Table 7). Remarkably, among these pathways, 3 sentinel genes, including *SELE*, *NOTCH3*, and *FGFR3*, emerged as being involved in at least 2 NPC-associated pathways and being associated with NPC in the gene-based test at the *P* < 1 × 10^–4^ level (Figure 2A). Particularly, *SELE* demonstrated the strongest association with NPC (*P_gene_* = 2.69 × 10^–6^) and was involved in the highest number of NPC-associated pathways (Figure 2A).

In the replication stage, we resequenced the exon regions of these 4 candidate genes (*RPL14*, *SELE*, *FGFR3*, and *NOTCH3*) using Cap-seq in our replication HK and GD-ZS cohorts. Metaanalyzed gene-based association tests across these replication datasets statistically affirmed the associations for all candidate genes (*P_Bonferroni_threshold_* < 0.01, *n_test_* = 5; Table 2), underscoring the pivotal roles these genes and their associated pathways play in NPC development.

We next fine-mapped the variants (MAF > 0.001, *n_SNVs_* = 58) contributing to the gene-level associations for the 4 replicated genes using the discovery dataset. A constituent variant was considered to have a “major” contribution if its variant-level association *P* value reached the α level of 0.01 significance after adjusting for 58 tests using the Bonferroni’s method (*P*_Bonferroni_threhold(α=0.01)_ < 1.72 × 10^–4^). This criterion led us to identify major contributions from a common variant in *RPL14* and several rare variants in *SELE* (Supplemental Tables 8 and 9). By contrast, no major contributing variants were detected for *FGFR3* or *NOTCH3* (Supplemental Tables 10 and 11).

Specifically, among the 4 SNVs in *RPL14*, the sentinel SNV rs2276868 (MAF = 0.36, *P_SNV_* = 4.16 × 10^–7^) and another SNV rs2276869 (*P_SNV_* = 0.001), which share modest linkage disequilibrium (LD) (*R^2^* = 0.2), were the only 2 variants associated with NPC in *RPL14* (*P_SNV_* < 0.05; Supplemental Table 8). Excluding these 2 SNVs from the gene-based analysis diminished the gene-level association of *RPL14* with NPC (*P_gene_* = 0.34). Additionally, controlling for rs2276868 also abolished the associations of rs2276869 (Supplemental Table 8). These findings suggest a predominant contribution of common variant rs2276868 to *RPL14*’s association with NPC.

In *SELE*, of the 23 SNVs identified, 10 were strongly associated with NPC in the single-variant test, led by rs3917410 (*P_SNV_* = 2.68 × 10^–5^), which shared nearly complete LD with the other 9 SNVs (*R^2^* > 0.99; Supplemental Table 9). Notably, all the 10 SNVs are rare (MAF < 0.01), with rs5361 being the only nonsynonymous variant (Figure 2B). Controlling for rs5361 abolished the association signals with NPC for the remaining 9 rare SNVs in *SELE* (*P_FDR_adjusted_* > 0.05; Supplemental Table 9). The rs5361 variant is a missense mutation (T>G) in the exon 4 of *SELE*, resulting in an aa substitution from the conservative serine (S) to arginine (R) at position 149 (Figure 2, B and C). This S149R substitution was predicted to be deleterious (Figure 2D). Collectively, these findings suggest that the rare variant rs5361 (*SELE*-S149R) is likely the causal variant driving *SELE*’s association with NPC.

### Genetic contributions of common and rare variants to NPC susceptibility.

**Figure 3:**
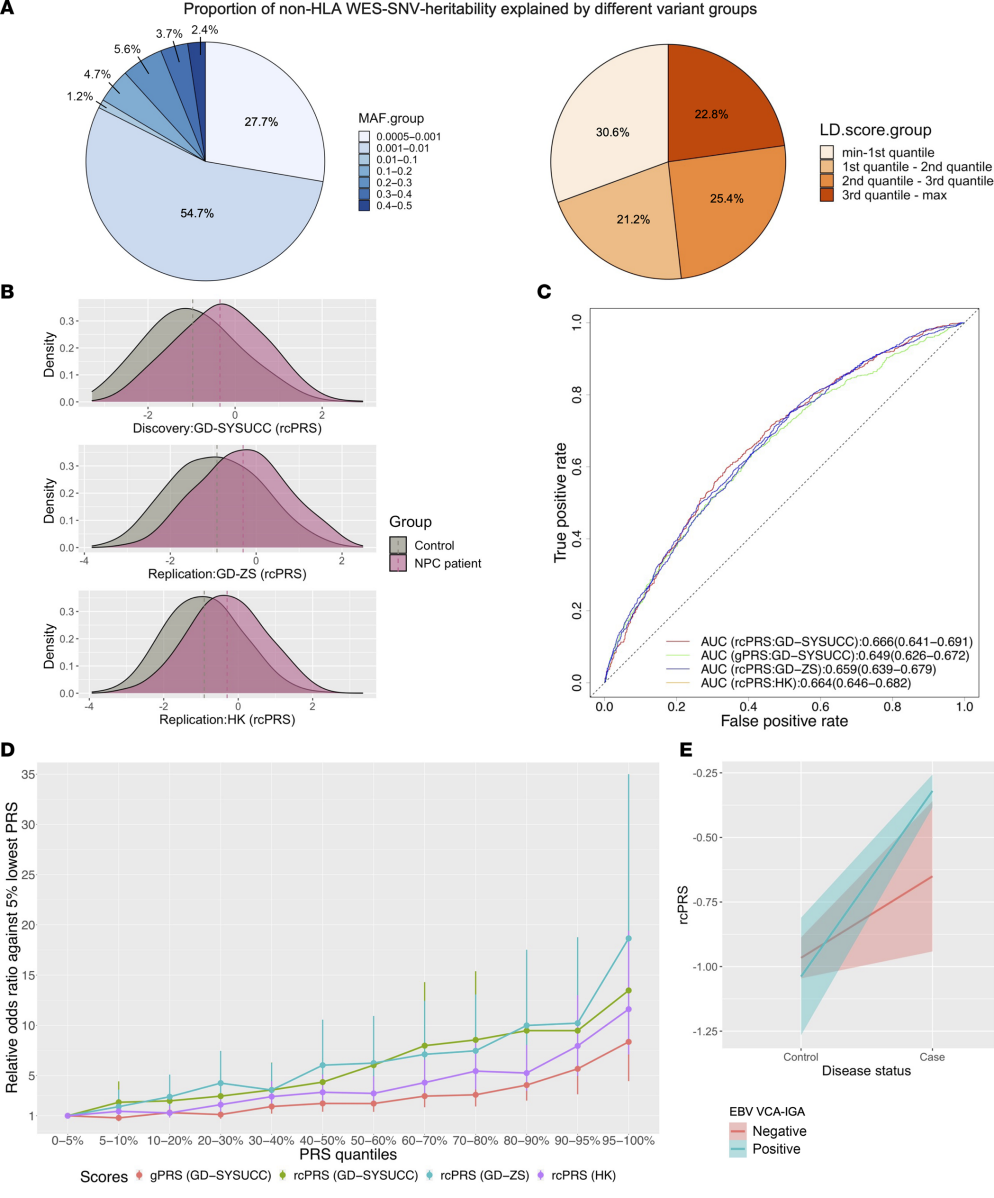
Contribution of common and rare variants to NPC susceptibility. (**A**). Fractional representation of NPC heritability attributable to non-HLA WES-SNVs, categorized by MAF and LD. For each variant, MAF was calculated using the discovery samples, and LD score represented the aggregated *R^2^* with adjacent variants spanning a 200 kb window. (**B**) Density plots illustrating the PRS incorporating the identified loci and previously known GWAS loci (rcPRS) for cases and controls in both the discovery and replication cohorts. (**C**) Receiver operating characteristic curves comparing the rcPRS and previously reported GWAS PRS (gPRS) for NPC across different cohorts. (**D**) Relative odds ratio comparing the rcPRS or gPRS bins and the 5% lowest quantile group in different cohorts. Stratification of individuals based on their NPC PRSs, either rcPRSs or gPRSs, revealed a more pronounced rising trend in the relative disease risk with the escalating rcPRS compared with the gPRS. (**E**) Correlation of rcPRS with disease status in individuals categorized by their EBV VCA-IgA status.

We evaluated the overall susceptibility of NPC attributable to SNVs identified through WES (see Methods). Our analyses revealed that 15.2% of NPC susceptibility (with a standard error of 3.6%) could be attributed to genetic effects from all WES-derived SNVs, including 4.4% linked to variants in the HLA region and 10.8% linked to those in the non-HLA regions. Notably, rare variants (MAF < 0.01) accounted for a substantial proportion (82.4%) of the WES SNV heritability in the non-HLA regions, among which the most independently acting variants (lowest LD scores) contributed the largest proportion (30.6%; Figure 3A).

Through a comparative analysis using a joint model to evaluate multiple genetic risk factors simultaneously (see Methods), we further discovered that the minor allele G of the rare *SELE*-rs5361 variant conferred a substantial disease risk in Guangdong datasets (GD-SYSUCC and GD-ZS; OR = approximately 2.20–2.22; Supplemental Figure 4). This risk level is comparable to that of the top 20% of the population’s polygenic risk score (PRS) derived from HLA variants (OR = approximately 2.14–2.30) and exceeds the risk associated with the minor allele T of the common *RPL14*-rs2276868 variant (OR = 1.22~1.28) or the top 20% of the population’s PRS for NPC derived from non-HLA GWAS variants (OR = 1.31-1.80; Supplemental Figure 4) within the same Guangdong datasets. These findings underscore the substantial genetic effect of rare variant on disease risk (11). Additionally, our estimation of the proportion of phenotypic variance explained by individual locus (see Methods) revealed that while HLA loci predominantly account for NPC’s phenotypic variance, the common *RPL14* variant explains a greater portion of disease variance than the rare *SELE* variant (Supplemental Figure 4), indicating the complex genetic architecture underlying NPC susceptibility (11).

### Polygenic risk prediction improved by identified loci and mediated by EBV infection.

To assess the predictive power of the identified genetic loci on NPC risk, we developed a refined composite polygenic risk score (rcPRS) that incorporates both NPC-associated SNVs and the leading SNVs from genes and pathways identified in this and previous studies (see Methods; Supplemental Table 12 and Figure 3B). Notably, the rcPRS demonstrated superior predictive performance, with an AUC ranging from 0.659 to 0.666 in the discovery and replication samples, outperforming the existing GWAS-based PRS (gPRS; AUC = 0.649; Figure 3C) (6). This improvement is largely attributable to the inclusion of the 2 identified common variants, rs1050462 in *HLA-B* and rs2276868 in *RPL14*, as highlighted by our leave-one-out analysis (Supplemental Table 13). Stratification analysis based on PRSs revealed a sharper increase in relative disease risk among individuals with higher rcPRSs, with those in the top percentile rage (95%–100%) showing a 1.6-fold increase in risk (OR_rcPRS_discov_ = 13.5) compared with those with higher gPRSs (OR_gPRS_discov_ = 8.4; Figure 3D). This indicates the improved capability of the rcPRS for more precise risk stratification in NPC.

It is noteworthy that in a focused analysis of a subset of 1, 018 cases and 774 controls from the GD-SYSUCC cohort, who had serological data for the EBV-encoded virus capsule antigen (EBV VCA-IgA), we observed a stronger association between rcPRS and NPC susceptibility in EBV seropositive individuals (Figure 3E). This finding emphasizes EBV’s role in modulating polygenic risk. Interaction analysis further confirmed that the polygenic risk in NPC is significantly mediated by serum EBV status (*P_interaction_* = 0.03), underscoring the greater utility of the rcPRS in predicting NPC risk, particularly in the context of serological EBV-positive populations.

### Distinct cellular expression patterns of NPC-associated genes.

**Figure 4:**
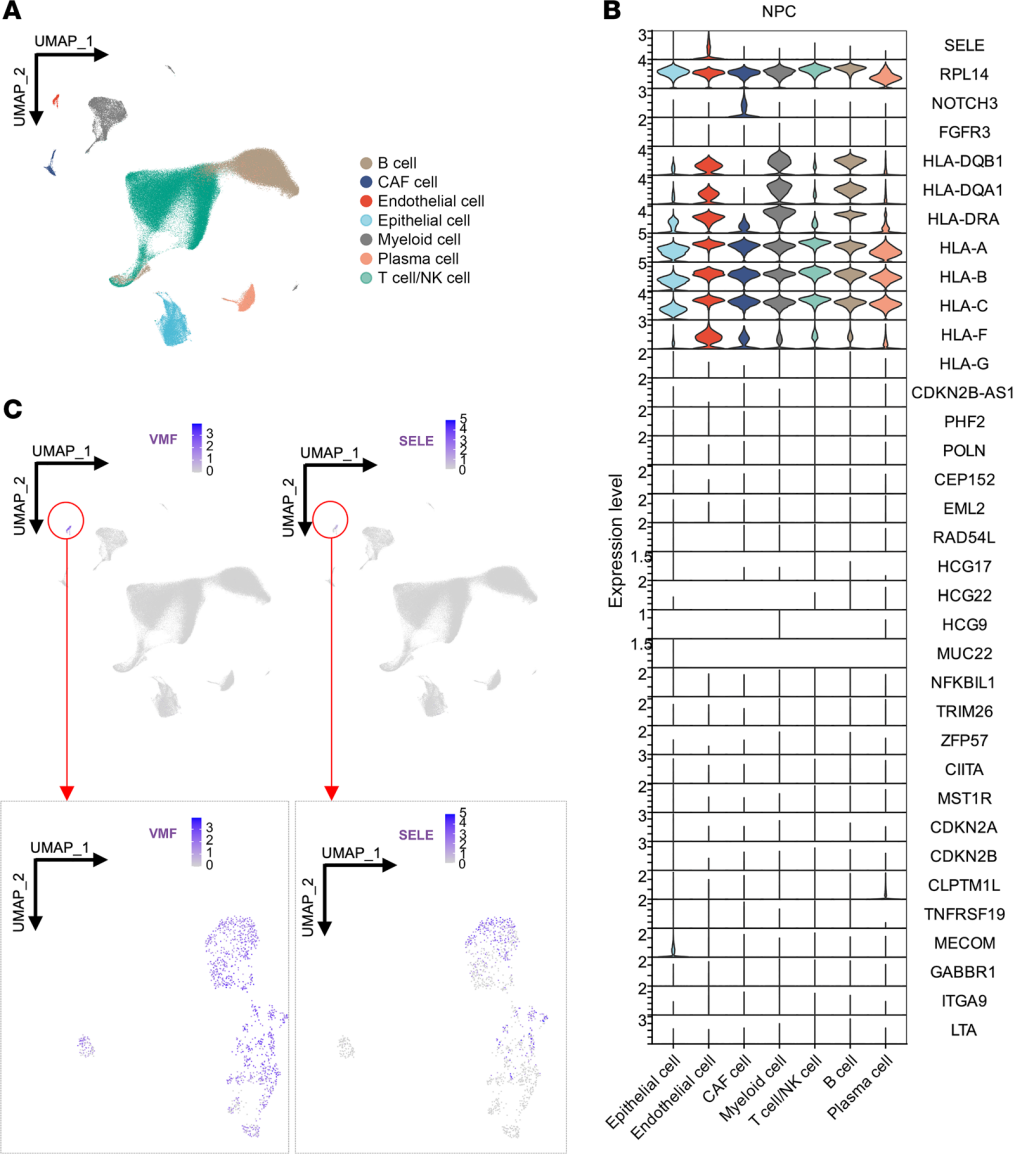
Expression patterns of identified and known NPC-associated genes across diverse cell types in NPC tumor tissues. Single-cell transcriptomic analyses of 223,593 cells derived from NPC tumor tissues (*n* = 56). (**A**) UMAP plot of 223,593 single cells grouped into 7 major cell clusters as indicated in the right panel. (**B**) Violin plot illustrating normalized expression of NPC-associated genes across the major cell clusters indicated at the bottom. All epithelial cells captured in NPC tumor were malignant (see Methods). (**C**) The expression of the marker gene (*VWF*) for endothelial cells, alongside the identified NPC-associated gene *SELE*; top panel: initial UMAP plot, bottom panel: renormalized UMAP emphasizing cells highlighted by the red circles in the initial UMAP plot. Each dot represents 1 cell, and color heatmap from white to purple represents expression level from low to high.

To understand the functional implications of the identified loci in NPC, we examined the cellular expression patterns of these loci along with known NPC-associated genes (Supplemental Table 14). Utilizing scRNA-Seq analysis of tumor samples from 56 NPC patients and nontumor tissues from 15 noncancerous donors (25) (see Methods), we found that *RPL14* was universally expressed across all cell types, particularly malignant epithelial cells (Figure 4, A and B). In contrast, *SELE* expression was specific to endothelial cells in both tumor and nontumor tissues (Figure 4C and Supplemental Figure 5).

Beyond *SELE*, other risk genes, such as *NOTCH3* and *HLA-II*, were predominantly expressed in stromal and immune cell types rather than in malignant epithelial cells within the TME (Figure 4). This expression pattern was consistently observed across other cancer types (Supplemental Figure 6), indicating that susceptibility genes are not necessarily confined to expression in malignant cells, but frequently exhibit predominant expression in diverse stromal or immune cell populations within the TME. These findings highlight the crucial role of genetic impacts on stromal cells in cancer development, challenging the traditional focus on gene expression solely within malignant cells.

### rs2276868 regulates RPL14 expression through the NKRF transcription factor.

**Figure 5:**
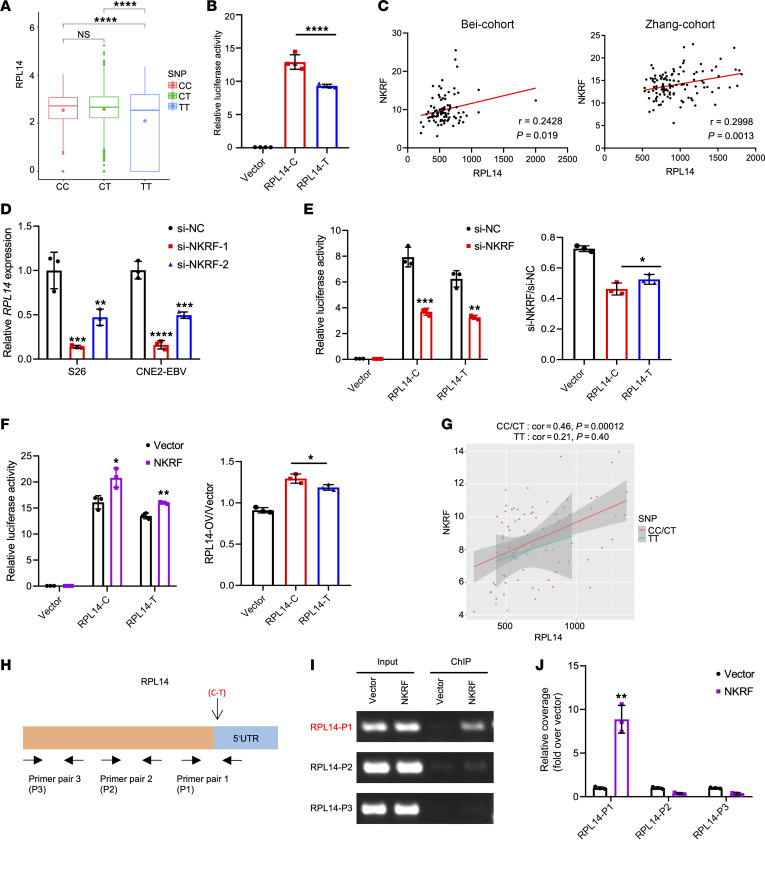
rs2276868 regulates the expression of *RPL14*. (**A**) Single-cell transcriptome analysis shows the mRNA expression of *RPL14* in 15,623 malignant epithelial cells from 35 NPC samples grouped according to their rs2276868 genotypes (CC, CT, or TT). (**B**) Relative luciferase activity changes in 293T cells transfected with plasmids containing rs2276868-[C], -[T], or control vectors. (**C**) Pearson’s correlation analysis indicates the relationship between *RPL14* and *NKRF* expression (measured as TPM) in transcriptome data of NPC patients from 2 cohorts, Bei-cohort (*n* = 93) and Zhang-cohort (*n* = 113). (**D**) RT-qPCR illustrates the mRNA expression of *RPL14* in NPC cells transfected with NKRF siRNAs or control siRNA. (**E** and **F**) Relative luciferase activity in 293T cells cotransfected with the rs2276868-[C] or -[T] plasmids and NKRF siRNA (**E**) or NKRF overexpression vectors (**F**). Corresponding statistics are presented at the right. (**G**) Pearson’s correlation analysis indicates the relationship between *NKRF* and *RPL14* expression in bulk RNA-Seq data are different for NPC patients with different genotypes. rs2276868-[CC/CT] patients have a stronger correlation than rs2276868-[TT] patients. (**H**) Schematic diagram indicates primer pairs used for PCR amplification of *RPL14* fragments. (**I** and **J**) ChIP assay in S26 cells transfected with Flag-NKRF and control vectors. ChIP PCR (**I**) and qPCR (**J**) analyze the binding of NKRF on rs2276868 at RPL14 promoter in cells. P1-3 denotes primer pairs targeting genomic regions shown in **H**. Between-group comparisons: *t* test for 2 groups, 1-way ANOVA followed by Šidák’s post hoc test (comparisons among all groups) or Dunnett’s post hoc test (comparisons with the control group) for 2 or more group comparisons. *P < 0.05; **P < 0.01; ***P < 0.001; ****P<0.0001.

Considering the location of rs2276868 at the promoter of *RPL14* (Supplemental Figure 7 and Supplemental Figure 8A) and the predominant expression of *RPL14* in malignant epithelial cells (Figure 4), we investigated rs2276868’s potential regulatory role in *RPL14* mRNA expression in epithelial cells. An scRNA-Seq–based expression quantitative trait loci (eQTL) analysis of 15,623 malignant epithelial cells from 35 NPC patients revealed that the CC or CT genotype of rs2276868 was significantly associated with higher *RPL14* expression compared with the TT genotype (Figure 5A). This was further corroborated by luciferase reporter assays, which showed reduced activity for the rs2276868-[T] construct (Figure 5B). Collectively, these findings strongly suggest that rs2276868 modulates *RPL14* transcription, likely by altering the binding affinity of specific transcription factors (TFs).

To identify the potential TFs involved in this regulation, we utilized 2 independent bulk transcriptomic datasets of NPC tissues (*n_Bei_* = 93 and *n_Zhang_* = 113) and identified 24 candidate TFs correlated with *RPL14* expression (Supplemental Figure 8B). Subsequent siRNA screening of these candidates pinpointed 5 TFs capable of modulating *RPL14* expression, with *NKRF* and *E2F5* being particularly notable, whose knockdown significantly reduced *RPL14* transcription in NPC cells (Figure 5, C and D, and Supplemental Figure 8, C, D, F, G). Further luciferase reporter assays demonstrated that only *NKRF* depletion substantially reduced the luciferase activity of the *RPL14* promoter (Figure 5E), whereas E2F5 knockdown exhibited opposite effects (Supplemental Figure 8E). *NKRF* also exhibited a differential impact on the regulatory function of rs2276868 variants, preferentially enhancing the transcription activity of the rs2276868-[C] construct (Figure 5, E and F). In bulk RNA-Seq data from NPC tumors, a stronger positive correlation between *NKRF* and *RPL14* expression was observed in patients carrying the rs2276868-[C] (Figure 5G). ChIP assays further confirmed NKRF’s specific binding to the genomic region encompassing rs2276868 (Figure 5, H–J, and Supplemental Figure 8H). Together, these findings strongly suggest that rs2276868 regulates *RPL14* expression through its interaction with *NKRF*, which preferentially enhances *RPL14* transcription in NPC cells carrying rs2276868-[C].

### RPL14 suppresses EBV activities and tumorigenesis in NPC.

**Figure 6:**
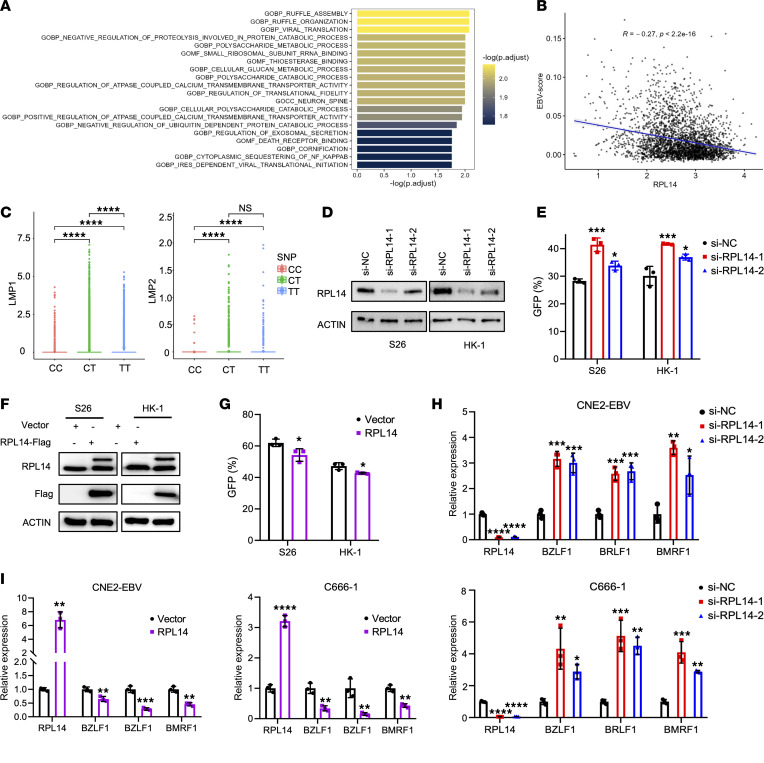
*RPL14* inhibits EBV infection and lytic cycle activation in NPC cells. (**A**) The top 20 pathways significantly associated with *RPL14* expression in malignant epithelial cells from NPC tumor. (**B**) Correlation analysis between *RPL14* expression and EBV-activity scores within malignant epithelial cells (dots). (**C**) Single-cell transcriptome analysis showing *LMP1* and *LMP2* expression in NPC samples with rs2276868-[CC], -[CT], or -[TT] genotypes. (**D**) Western blot assessment of the knockdown efficiency of RPL14 siRNAs or control siRNA in S26 and HK-1 cells. Actin was used as a loading control. (**E**) Flow cytometry quantification of GFP intensity for the EBV infection efficiency in the NPC cells described in **D**. (**F**) Western blot assay showing RPL14 protein expression in S26 and HK-1 cells infected with lentivirus stably expressing RPL14. Actin served as a loading control. (**G**) Flow cytometry assessment of EBV infection efficiency in the cells described in **F**, which was then infected with EBV. (**H** and **I**) RT-qPCR analysis of EBV lytic gene expression in CNE2-EBV and C666-1 cells transfected with RPL14 siRNAs or control siRNA (**H**) or infected with lentivirus stably expressing RPL14 vector or control vector (**I**). Between-group comparisons: *t* test for 2 groups, 1-way ANOVA followed by Šidák’s post hoc test (comparisons among all groups) or Dunnett’s post hoc test (comparisons with the control group) for comparisons among more than 2 groups. *P < 0.05; **P < 0.01; ***P < 0.001; ****P < 0.0001.

To explore the role of *RPL14* in NPC development, we conducted pathway analyses to examine the relationship between *RPL14* expression and the transcriptional activity of molecular pathways in NPC tumor tissues, employing the scRNA-Seq and bulk RNA-Seq data (see Methods). These analyses revealed significant associations between *RPL14* and several pathways crucial to viral processes (Figure 6A, Supplemental Figure 9, and Supplemental Tables 15–17) in NPC. Considering the well-known involvement of EBV in NPC etiology (26), we hypothesized a potential link between *RPL14* and EBV activities. Supportively, we observed a significant negative correlation (*R*= –0.27, *P* < 2.2 × 10^–16^) between *RPL14* expression and EBV activity score in EBV-positive malignant NPC cells (see Methods; Figure 6B). This inverse relationship was particularly pronounced among rs2276868-[T] carriers, who showed reduced *RPL14* expression alongside increased expression of EBV latent genes *LMP1* and *LMP2* as compared with rs2276868-[C] carriers (Figure 6C). These results suggest that *RPL14* suppresses EBV-driven processes.

**Figure 7:**
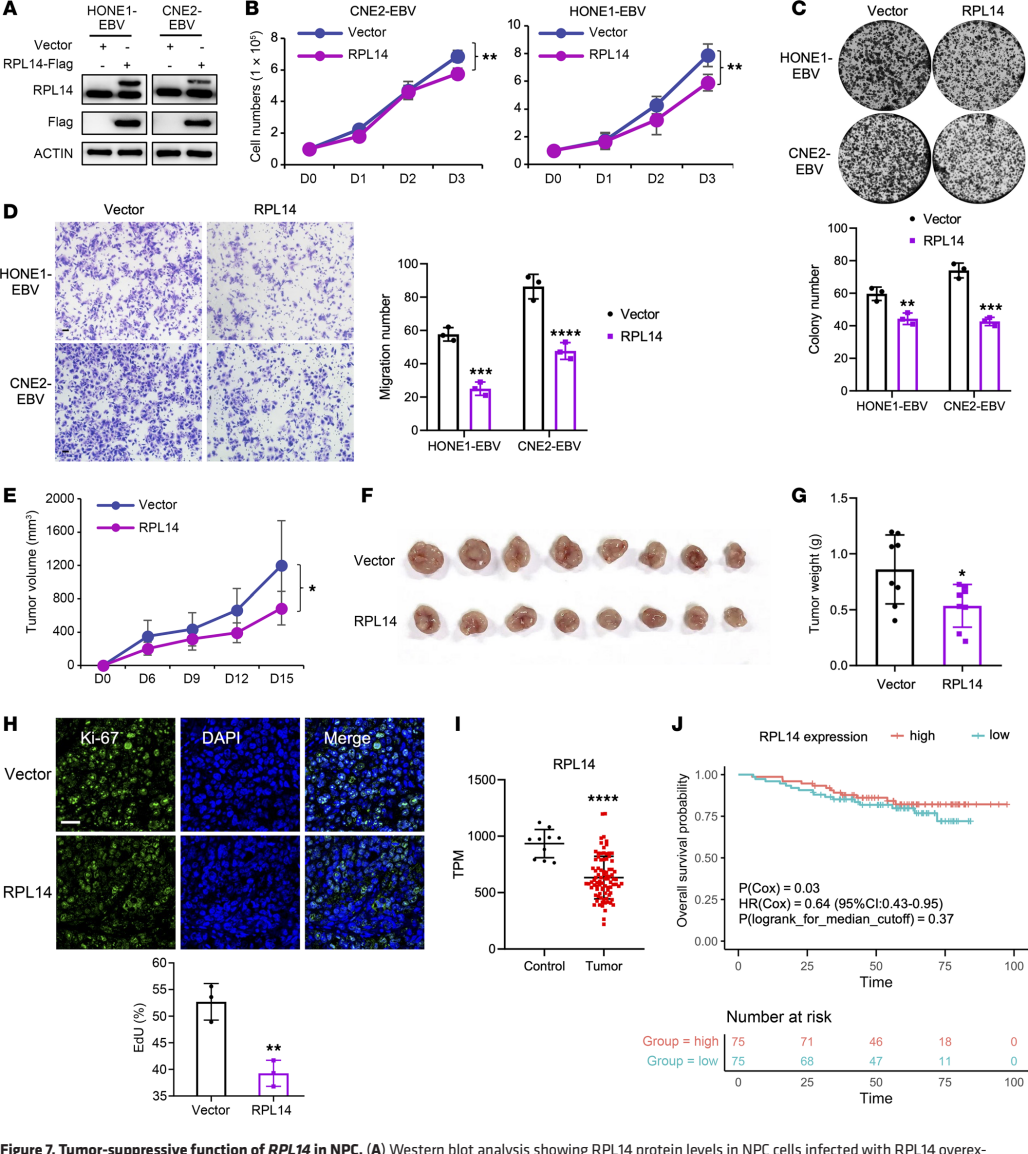
Tumor-suppressive function of *RPL14* in NPC. (**A**) Western blot analysis showing RPL14 protein levels in NPC cells infected with RPL14 overexpressing lentivirus. Actin serves as the loading control. (**B**) Cell growth curves of the cells described in **A**. (**C**) Colony formation assay for the cells described in **A**. Corresponding statistical analysis is shown below. (**D**) Transwell migration assay evaluating the migration ability of the cells described in **A**. Scale bar: 50 μm. The statistical analysis is presented on the right. (**E**–**H**) Tumor growth evaluation in a nude mouse model with subcutaneous injection of CNE2-EBV cells described in **A**. Tumor volumes were measured every 3 days. Visual presentation of tumor after sacrifice (**F**) and weight (**G**) were presented. IF detection of Ki-67 expression (**H**) in the tumors described in **F**, and the corresponding statistical analysis is shown on the right. Scale bar: 50 μm. (**I**) Transcriptomic analysis showcasing mRNA levels of *RPL14* in NPC (*n* = 87) versus control samples (*n* = 10). (**J**) Kaplan-Meier survival curve and Cox’s regression analyses linking *RPL14* expression to overall survival (OS) of NPC patients in the Chen et al. cohort (*n* = 150). *RPL14* expression levels were adjusted using *EPCAM* expression to account for the epithelial cell proportion in tumor tissue and subsequently scaled to a mean of 0 and a variance of 1. *P*(Cox) and HR(Cox) represent the *P* value and hazard ratio for the effect of *RPL14* expression on OS in the Cox-regression model, adjusting age and sex. 95%CI: 95% confidence interval. *P*(log-rank): *P* value from the log-rank test comparing 2 groups with high (red) versus low (blue) *RPL14* expression, determined by the median in the Kaplan-Meier analysis. Statistical method for between-group comparisons: *t* test. *P < 0.05; **P < 0.01; ***P < 0.001; ****P < 0.0001.

Further investigation into *RPL14*’s regulatory role in EBV activities showed that RPL14 knockdown significantly increased EBV infection in NPC cells, while its overexpression led to a marked decrease (Figure 6, D–G). Additionally, RPL14 knockdown substantially enhanced the expression of EBV lytic genes in NPC cells, whereas RPL14 overexpression significantly reduced their expression (Figure 6, H and I), reinforcing the suppressive role of RPL14 in both EBV infection and lytic cycle in NPC. These observations led us to hypothesize that *RPL14* may contributes to the suppression of EBV-related tumorigenesis. Indeed, in EBV^+^ NPC cells, cell growth curves and colony formation assays demonstrated that *RPL14* overexpression inhibited NPC cell proliferation (Figure 7, A–C), while its knockdown promoted proliferation (Supplemental Figure 10, A–C). Transwell assays further showed that *RPL14* overexpression suppressed NPC cell migration, whereas knockdown enhanced it (Figure 7D and Supplemental Figure 10D). In an in vivo mouse model with subcutaneous injection of RPL14-expressing NPC cells, RPL14 significantly inhibited tumor growth, as evidenced by reduced tumor volume and weight (Figure 7, E–G). Supportively, immunofluorescence staining revealed fewer proliferating cells in the *RPL14*-overexpressing tumors (Figure 7H). Additionally, lower *RPL14* expression levels were observed in NPC tissues compared with control samples (Figure 7I) and were associated with worse overall survival in NPC patients from an independent cohort (*n* = 150; *P_Cox_* = 0.03; HR_Cox_ = 0.64, 95%CI = 0.43–0.95; Figure 7J and Supplemental Figure 11) (27). Collectively, these findings underscore *RPL14* as a pivotal suppressor for EBV and tumor in NPC.

### SELE-S149R promotes NPC development through gain of function in endothelial cells.

**Figure 8:**
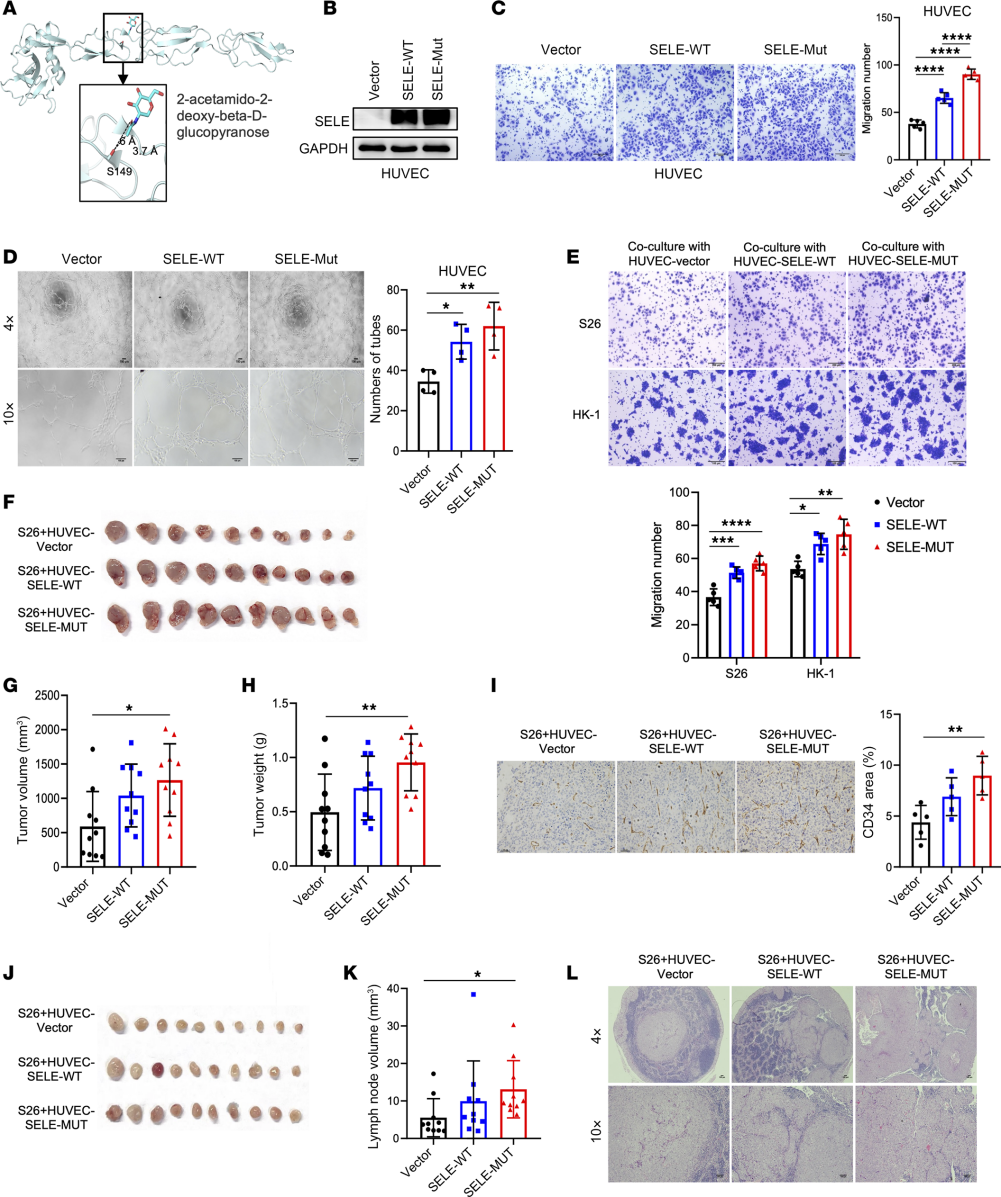
SELE-S149R mutation in endothelial cells promotes the tumorigenesis and metastasis of NPC cells. (**A**) Structure prediction of E-selectin paired with glycomimetic antagonist ligand 2-acetamido-2-deoxy-beta-d-glucopyranose (PDB code 4C16). The spatial proximity between Ser149 and the ligand is highlighted. Both the Ser149 side chain and the ligand are shown as sticks, with the distances between the OG atom of Ser149 and the C8 or O7 atoms of the ligand specified. (**B**) Western blot examination of SELE protein level in HUVEC cells infected with lentivirus overexpressing SELE-WT, S149R mutant, or control vectors. (**C**) Transwell migration assay evaluating the migration ability of the cells described in **B**, with statistical analysis presented to the right. Scale bar: 100 μm. (**D**) Tube-formation assay with cells described in **B**. The statistical analysis is presented on the right. Scale bars: 100 μm. (**E**) Transwell migration assay assessing the migration ability of NPC cell lines (S26 and HK-1) cocultured with HUVEC cells from **B** (S26/HK-1: HUVEC = 10:1). The statistics are presented at the bottom. Scale bars: 200 μm. (**F**–**H**) Tumor growth evaluation in xenograft model with subcutaneous injection of S26 cells described in **E**. Tumor volumes (**G**) are measured; visual presentation (**F**) and weight (**H**) of tumor after sacrifice are presented. (**I**) Representative image for IHC staining of CD34 in tumors presented in **F**. The statistics are presented on the right. Scale bars: 100 μm. (**J**–**L**) Tumor lymphatic metastasis of S26 cells described in **E**. Lymph nodes are visualized (**J**) and measured (**K**), with H&E staining conducted to assess metastasis in these lymph nodes, of which representative image is shown (**L**). Scale bar: 100 μm. One-way ANOVA followed by Šidák’s post hoc test was applied to comparisons among all groups or Dunnett’s post hoc test was applied to comparisons with the control group for comparisons among more than 2 groups. *P < 0.05; **P < 0.01; ***P < 0.001; ****P<0.0001.

E-selectin, encoded by *SELE*, is a crucial cell-adhesion molecule specifically expressed on the surface of endothelial cells (Figure 4C), particularly attracting cancer cells by interacting with glycoproteins (28). Structural modeling of E-selectin paired with the glycomimetic antagonist ligand revealed that the S149R substitution, resulting from the rs5361 variant (Figure 2B), extends E-selectin’s side chain (Figure 8A), potentially modifying its interaction with ligands and conferring a gain-of-function capability. This finding aligns with a previous finding that *SELE-*S149R enhances the recruiting capability of E-selectin (29).

To probe the functional implications of the *SELE-*S149R mutation, we utilized human umbilical vein endothelial cells (HUVECs) engineered to stably overexpress either the WT *SELE* (*SELE*-Ser149, SELE-WT) or its S149R mutant variant (SELE-MUT, Figure 8B). Transwell assays revealed that the SELE-MUT significantly increased HUVEC migration (Figure 8C). Furthermore, tube-formation assay demonstrated that the SELE-MUT markedly enhanced HUVEC’s ability to form capillary-like structures (Figure 8D), indicating that the S149R mutation potentiates angiogenesis. In a coculture model with NPC cells, HUVECs overexpressing SELE-MUT substantially increased NPC cell migration (Figure 8E). This observation was further substantiated by an in vivo xenograft model, where NPC cells cocultured with HUVEC-SELE-MUT exhibited increased tumorigenesis, as evidenced by augmented tumor volume and weight (Figure 8, F–H) along with enhanced angiogenesis as indicated by CD34 staining indicative of vessel formation (Figure 8I). Additionally, lymph node metastasis assays reinforced these findings, showing that coculture with SELE-MUT HUVECs significantly promoted NPC cell metastasis (Figure 8, J–L). These results underscore the critical role of *SELE-*S149R mutation in driving NPC tumorigenesis and metastasis by enhancing endothelial cell function.

## Discussion

Here we conducted the most extensive WES association study on NPC to date, uncovering 3 genetic variants and genes linked to NPC susceptibility, including the common variant rs2276868 in *RPL14*, the rare coding variant rs5361 in *SELE*, and the common variant rs1050462 within the HLA-B locus. The replication of these associations in additional and independent GD-ZS and HK cohorts, which share a close genetic background but have experienced distinct industrialization and lifestyle westernization, underscores the robustness of these genetic effects on NPC susceptibility, irrespective of varying environmental exposures. The discovery of the *RPL14* locus can be attributed to the large sample size and the more pronounced genetic risk observed in familial cases (OR_FHNPC_ = 1.575) compared with sporadic cases (OR_sporadic_NPC_ = 1.200), a pattern consistent with other complex diseases (30). The identification of *SELE* underlines the effectiveness of our approach in dissecting disease associations at the pathway level. Notably, our study indicates that the predictive performance of the rcPRS for NPC is influenced by the serological status of EBV activation, suggesting that polygenetic risk in NPC can be modified by internal environmental factors, such as EBV. Considering that both EBV DNA load and antibodies against EBV are established serological markers for early NPC diagnosis (16), incorporating the rcPRS with EBV serological markers could substantially improve personalized risk assessments, early detection, and treatment outcomes.

Our study sheds light on the functional impact of the identified genes on NPC, enhancing our comprehension of its pathogenic mechanisms. We found that the NPC-associated variant rs2276868 modulates *RPL14* expression through the TF *NKRF*, an inhibitor of the NF-κB pathway involved in NPC development, (31). Our findings further demonstrate that *RPL14* suppresses the EBV life cycle in NPC, consistent with the reported role of other RPL family members in EBV regulation (32). Given the critical role of uncontrolled EBV activity in NPC development (33), it is plausible that *RPL14*, with its ability to inhibit EBV activity, plays a key role in repressing tumorigenesis. Indeed, our in vivo and in vitro assays showed that *RPL14* is highly effective in regulating NPC tumorigenesis in EBV-positive cells, consistent with a previous report using in vitro assays (34). Collectively, these findings suggest that the pathological consequences of varied *RPL14* expression are likely mediated through its regulatory role in the EBV life cycle. Therefore, targeting *RPL14* expression and EBV activities may represent potential therapeutic strategies for NPC, especially considering that inhibitors of downstream signaling pathways altered by ribosomal defect or ribosome biogenesis have been implicated in human diseases (35).

Our study also identifies *SELE* as an NPC-associated gene, featuring the rare germline mutation rs5361 in its encoding cytoadhesive glycoprotein E-selectin. E-selectin is known for its interactions with various cells, including leukocytes, cancer-associated fibroblasts, and malignant epithelial cells (28, 36, 37). This mutation, leading to a deleterious S149R substitution, enhances the functionality of E-selectin in endothelial cells, promoting their rolling and adhesion capabilities (29). Our study demonstrates that *SELE* expression is confined to endothelial cells in tumor tissues and that endothelial cells expressing SELE, particularly those with the S149R mutation, substantially render stronger migration and tumorigenesis to malignant NPC cells. This highlights the crucial role of E-selectin in mediating interactions with cancer cells and modulating metastasis in various cancers (28, 38). Together with previous findings that *SELE* and its S149R mutation can serve as a prognostic marker for colon cancer (39), our results suggest that the *SELE-*S149R may likely enhance the adhesion properties of E-selectin in endothelial cells, reshaping the key TME component to support cancer progression (38), thereby affecting individual susceptibility to NPC. Given that a phase 1/2 clinical trial has demonstrated the addition of the E-selectin antagonist uproleselan (GMI-1271) to chemotherapy is well tolerated and associated with high remission rates and improved survival in acute myeloid leukemia (40), incorporating E-selectin antagonists into chemotherapy might offer a promising therapeutic option for NPC.

EBV is a crucial TME component in EBV-related malignancies (18, 41). Our findings, which establish a functional link between the NPC-associated variant rs2276868, *RPL14* expression, and EBV activity, reinforce the notion that genetic variations affecting EBV-related tumorigenesis contribute to varied susceptibility to NPC (42). Furthermore, our study unveils the specific expression patterns and functions of *RPL14* and *SELE* within distinct effector cell types (epithelial cells and stromal endothelial cells, respectively) in NPC. Importantly, the diverse expression of risk genes within different TME cell components is consistent across other NPC-associated genes and other cancer types (Supplemental Figure 6). These observations highlight the complex roles that germline variations play across diverse cellular contexts in cancer susceptibility, resonating with a growing body of literature suggesting the engagement of various cell types in the development of complex disease (43). This stands in contrast to traditional approaches that primarily focus on cancer-originating cells (e.g., epithelial cells in NPC) when validating the functional relevance of genetic susceptibility genes. Our findings thus challenge this conventional perspective, advocating for the necessity of broadening investigations into how cancer risk genes function across various TME components in cancer development (such as pathogens and endothelial cells).

In summary, our study addresses the missing heritability of NPC and advances our understanding of NPC’s genetic architecture by unveiling multiple genetic associations across both common and rare variants. Importantly, our innovative rcPRS model incorporates these discoveries and reflects the modifying effect of EBV status on polygenic risk, providing a more targeted screening solution for NPC. Moreover, our findings shed light on a spectrum of pathogenic mechanisms, particularly highlighting the role of EBV-mediated tumorigenesis via *RPL14* in malignant epithelial cells and the influence of *SELE* on the adhesive properties of endothelial cells. Our study underscores the profound impact of genetic variants on the diverse TME components, which jointly contribute to NPC development. We acknowledge certain limitations, such as the sample size constraints, particularly concerning the familial NPC (FHNPC) group and the analysis of rarer variants, the need to validate our findings in non-Chinese ethnic groups, and the necessity of further studies to delve into the mechanistic links between these genetic variants and NPC pathogenesis. While our study aims to identify genetic risk loci, we recognize that future prospective studies integrating with conventional environmental and clinical factors will be crucial to optimizing the clinical applicability of genetic findings in NPC management.

## Methods

### Sex as a biological variable

Our study examined male and female humans, and similar findings are reported for both sexes.

### Patient recruitment and sample preparation

#### Discovery stage.

The discovery dataset consisted of 2,694 NPC cases (2,125 from the GD-SYSUCC and 569 from the SG) and 2,328 healthy controls (1,068 from the GD-SYSUCC and 1,260 from SG) of Chinese Han ancestry. Detailed information of the sample collections is described in Supplemental Note 1. Serum IgA antibodies to EBV capsid antigen (IgA-VCA) were available for 1,646 NPC patients and 918 controls in the GD-SYSUCC subset. Individuals with IgA-VCA of 1:20 or greater were categorized as EBV positive.

#### Replication stage.

The replication dataset included 2 cohorts: a Zhongshan cohort (GD-ZS) consisting of 1,941 cases and 2,265 controls from Zhongshan city in Guangdong province, and a HK cohort consisting of 2,334 cases and 2,507 controls from HK. Details of the sample collections are described in Supplemental Note 1. We considered the GD-ZS as a Guangdong indigenous cohort that shared regional demographic features with the discovery GD-SYSUCC cohort collected from the same province, whereas the HK cohort shared ancestral genetics with other cohorts but was relatively demographically different.

#### Bulk tissue samples for RNA-Seq and microarrays.

Tumor biopsy samples from 93 NPC patients were collected for bulk RNA-Seq (labeled as the “Bei” cohort). We also retrieved a published bulk RNA-Seq dataset for tumor tissues from 113 independent NPC patients that were collected from a published study (the “Zhang” cohort) (44). For survival analysis, we further retrieved a transcriptomic dataset generated using microarray for 150 NPC tumor tissues, together with prognostic data and relevant covariates (the “Chen et al. cohort”) (27). Additionally, we collected nasopharyngeal samples from 10 cancer-free individuals with nasopharyngeal inflammation at SYSUCC, using them as controls. Biopsies were collected before any treatment and immersed in RNAlater.

#### NPC samples for scRNA-Seq.

Tumor biopsies of 10 NPC patients were collected for scRNA-Seq in our previous study (18). Among them, 8 had tumor epithelial cells successfully captured. By extracting data from 4 additional studies (19, 45–47), we further collected scRNA-Seq data of 46 NPC tumor samples, 27 of which captured tumor epithelial cells (19, 45–47). In total, scRNA-Seq datasets consisting of 56 samples (of which 35 had epithelial cells captured) were used in downstream analyses.

### Data generation, quality control and annotation

#### WES and variant calling.

Whole blood DNA sample was extracted from all participants in the discovery dataset for WES. A library was constructed using one of the following three products: SureSelect Human All Exon V6+UTR kit (Agilent), SureSelect Human All Exon V5+UTR kit (Agilent), and SeqCap EZ Exome + UTR Target Enrichment Kit (Roche). This was followed by next-generation sequencing using a paired-end 2 × 150 bp protocol on an Illumina HiSeq instrument. Details of library construction, quality control, variant calling (including HLA genotyping), and variant annotation are described in Supplemental Note 2.

#### Cap-seq for targeted genomic regions and variant calling.

The selection criteria for the loci in the validation phase were all statistically significant findings at the discovery stage, including SNV level (index SNVs), genes, and pathway levels (leading genes of the pathway), as well as variants included in the rcPRS model. Genotyping was done by using the capture-based sequencing technology, where probes were designed to capture DNA fragments covering these SNVs and exon regions of these genes, followed by next-generation sequencing. Details of library construction and sequencing process are described in Supplemental Note 3.

#### Whole transcriptomic sequencing.

For bulk RNA-Seq, total RNA was extracted from tissue or cell line using the RNeasy Mini Kit (QIAGEN), with ribosomal RNAs removed by the Ribo-Zero Magnetic Kit (Illumina). The TruSeq RNA Library Prep Kit (Illumina) was used to construct the library, followed by RNA-Seq. RNA-*s*eq was conducted with a pair-end of 150 bp protocol on an Illumina Hiseq X sequencer. Bowtie was used to map post-QCed reads to the human reference genome (hg19), and Htseq was used to quantify reads counts (43). Per-gene expression level was normalized using transcripts per million (TPM). scRNA-Seq was performed following the manufacturer’s instructions (10x Genomics single-cell 5′ sequencing and 3’ sequencing kits). Details are described in previous studies (18, 19, 45–47).

### Analyses

#### Single-variant–based association test.

In the discovery stage, for variants in the non-HLA region, SNVs with a MAF of 0.001 or more were included in a logistic regression analysis in which NPC disease status was regressed on the genotype of individual SNV. For variants in the HLA region, 4 types of variants were included in analysis: SNVs, 4-digit HLA alleles, 6-digit HLA alleles, and aa. For each variant, 2 tests were performed for binary phenotypes: all NPC cases (ALLNPC) versus controls and NPC cases with family history (FHNPC) versus controls. Other covariates in the model included top 5 genetic PCs and sex. Top 5 PCs were chosen as this already secured a quite conservative EWAS, with the genomic inflation factor (λ) of 1.07 for the comparison between ALLNPC and control and 0.89 for the comparison between FHNPC and control. Bonferroni’s method was applied in multiple testing correction (*n* = 157,024) with a significance threshold *P* value of 3.2 × 10^–7^.

In the replication stage, significant associations identified in the discovery stage were tested in each of the replication cohorts. Logistic regression analysis was conducted, adjusting sex as a covariate. Bonferroni’s method was applied in multiple testing corrections (*n_tested_variants_* = 4), with a significance threshold *P* value of 0.0125.

#### Conditional analysis for single-variant–based associations.

To test whether the single-variant association from a target SNV was independent of the associations of other SNVs, conditional analysis was performed by jointly fitting the target SNV as a covariate variable in the regression model.

#### SNV-set–based association analysis 1: gene-based test.

The gene boundary was defined by the transcription start and end sites. These was no MAF-based filtering for this analysis (including singletons). We performed tests for 2 SNV types: all SNVs (SNV type 1 or ALL_SNV) or coding-affecting SNVs only (SNV type 2 or CODING_SNV). For each SNV type, we applied 4 algorithms in the “SKAT” package, including the original SKAT (48), the original Burden test (49), and these 2 tests inclusive of common and rare variants (detailed in Supplemental Note 4).

For each gene tested using each SNV type for each phenotype, the result from the algorithm with the most significant *P* value was kept. In the discovery stage, top 10 genetic PCs and sex were included in the model as covariates. Bonferroni’s method was applied in multiple testing corrections (*n_tested_gene_* = 22,228) with a significance threshold *P* value of 2.3 × 10^–6^. In the replication stage, significant association identified in the discovery stage was tested in each of the replication cohorts (*n_tested_gene_* = 1), adjusting sex as a covariate.

#### SNV-set–based association analysis 2: pathway-based test.

The pathway information was downloaded from the Molecular Signature Database (https://www.gsea-msigdb.org/gsea/msigdb), including curated canonical pathways, Gene Ontology (GO) items, and oncogenic signature gene sets (50). After annotating SNVs to the pathways, we performed pathway-level association analysis to test cumulative effect from both rare and common variants within a pathway on NPC risk. To avoid false-positive results driven by very small or very large gene sets, we excluded pathways with a size larger than 200 genes or smaller than 5 genes. The same grouping and algorithms applied in the above gene-based test were also applied here. In the discovery stage, top 10 genetic PCs and gender were included in the model as covariates. Multiple testing correction was performed using the FDR method (*P_FDR_threshold_* < 3.083 × 10^–4^; *n_tested_pathway_* = 6,204). In the replication stage, sentinel genes that were both involved in at least 2 significant pathways and obtained a gene-based *P* value lower than 1 × 10^–4^ in the discovery stage were tested for gene-level association in each of the replication cohorts (*n_gene_* = 3), adjusting sex as a covariate. Bonferroni’s method was applied in multiple testing correction.

#### Metaanalysis for single variant–based and gene-based associations.

The random effect model (REML) was applied for metaanalysis to combine the results of independent association analyses between individual variants and NPC risk using the R package “metafor” (51). The summation of logits method (52) was applied to metaanalyzing the independent association results between individual genes and NPC using the R package “metap” (53). I^2^ was used to assess the between-study heterogeneity (54).

#### Structural modeling of E-selectin.

The molecular structure of E-selectin complexed with glycomimetic antagonist 2-acetamido-2-deoxy-beta-d-glucopyranose (PDB code 4C16) was modeled as previously described (55). PyMOL with the Adaptive Poisson-Boltzmann Solver plugin was used for evaluating the electrostatic surface and visualization.

#### Assessing cell-type–specific expression of cancer-associated genes using scRNA-Seq data.

We summarized the susceptibility genes for NPC identified in the present study and those reported previously for NPC, lung, colorectal, and gastric cancers by GWAS studies (Supplemental Table 14). Gene-expression profiles were retrieved from multiple scRNA-Seq datasets, including the NPC dataset as described in the *NPC samples for scRNA-Seq* section, datasets for lung, gastric, and colorectal cancers, and the noncancer tissue dataset consisting of 10 tissues of 15 donors (25). The origins of these data are provided in Supplemental Note 1. Details of single-cell analyses including gene expression quantification, cell-type annotation, and inference of malignant epithelial cells are described in Supplemental Note 5. Normalized expression of marker genes for each cell type in each tumor datasets are shown in Supplemental Figure 12. For the noncancer tissue dataset, cell clusters were determined by the original study (25).

#### Estimate of WES-SNV heritability for NPC.

GREML-LDMS (the LD and MAF stratified genome-based restricted maximum likelihood [GREML]) approach was applied to estimate the proportion of NPC risk that can be explained by WES SNVs in different MAF and LD categories (56). Details in SNV groupings are described in Supplemental Note 6.

#### Joint model for a comparative analysis to estimate the relative risks by genetic variants.

We compared the relative risk of genetic effect from different variants in *RPL14*, *SELE*, HLA, and non-HLA GWAS loci, using a joint regression model. Detailed procedures are described in Supplemental Note 7 and Supplemental Table 18.

#### Phenotype variance explained by individual locus.

We estimated the proportion of phenotypic variance explained by each of the associated loci using a preestablished formula (57).

#### Construction and evaluation of PRS for NPC.

We calculated rcPRS for all individuals with nonmissing genotypes in the discovery GD-SYSUCC sample (the subset of 1,382 cases and 912 controls with both GWAS array and WES data available; ref. 7) and in all replication samples (with the Cap-seq data for the constituent variants of rcPRS). SNVs of multi-sources were selected to calculate rcPRS, simultaneously considering genetic loci identified in the present study and published GWASs (5, 7). Selection criteria and details of the constituent variants are described in Supplemental Note 8 and Supplemental Table 12.

To calculate gPRS for individuals in the discovery GD-SYSUCC sample, we imputed missing genotypes using the original BeadChips array data and constructed gPRS using NPC-associated loci reported by GWAS (6), following the instructions in the original study (6). Details of the imputation and PRS calculation are described in Supplemental Note 8.

#### Construction of composite scores for pathway and EBV activity.

We applied the AddModuleScore function in R package Seurat (58) to profile each pathway from the REACTOME and the GO Ontology database for each sample, where bulk RNA-Seq or scRNA-Seq data were available. Using the same function, we additionally created composite scores for EBV activity. Details are described in Supplemental Note 9.

#### The correlation of expression between RPL14 and other genes/pathways.

Pearson’s correlations between *RPL14* expression and pathway composite scores or EBV genes were tested using the cor.test function in R. Multiple testing correction was performed using the FDR method. Pearson’s correlations of *RPL14* and individual EBV genes are shown in Supplemental Table 19.

#### Survival analysis.

Cox’s proportional hazards model was applied with age and sex fitted as covariates to estimate the HRs and 95% CIs of *EPCAM*-normalized *RPL14* expression (scaled to a mean of 0 and a variance of 1). the Kaplan-Meier method was applied for sensitivity analyses comparing *RPL14* expression groups under various quartile-based cutoffs.

### Functional characterization of NPC-associated loci

#### Cell culture.

Human NPC cell lines S26, CNE2-EBV, and HONE1-EBV were gifts from Mu-Sheng Zeng at the SYSUCC. Human NPC cell lines HK-1 and C666-1 were gifts from K.W. Lo at the Chinese University of Hong Kong. Human embryonic kidney cell 293T cell was purchased from the Cell Bank of Type Culture Collection of the Chinese Academy of Sciences. All of the above cells were cultivated in DMEM medium containing 10% FBS (Gibco, Thermo Fisher Scientific) and 1% penicillin-streptomycin solution (FBS, Gibco, Thermo Fisher Scientific). HUVECs were isolated and cultured in ECM medium (ScienCell) supplemented with 15% FBS and 0.03 mg/mL of endothelial cell growth supplement (ECGS) (ScienCell). All cells were cultured at 37°C under a humidified atmosphere with 5% CO_2_. For all cell lines, mycoplasma was routinely determined using PCR with specific primers and no contamination was detected (Supplemental Table 20).

#### Cell proliferation.

For cell growth curves, a total of 1 × 10^5^ NPC cells were plated in a 12-well plate with 3 replicates. The number of cells was measured by a cell counter with trypan blue staining at 24, 48 and 72 hours. For colony-formation assays, NPC cells were seeded at a density of 3,000 cells per replicate into 6-well plate for 7–10 days. Then, colonies were fixed with 4% paraformaldehyde for 30 minutes at room temperature. After being stained with crystal violet for 10 minutes, cells were photographed with the Bio-Rad ChemiDoc Touch.

#### Cell migration.

Transwell assay was performed as described previously (59). Briefly, NPC cells in serum-free medium were plated at a density of 6 × 10^4^ cells into the transwell chamber (8 μm pores, Corning), which were then placed in 24-well plates containing 600 μL DMEM supplemented with 10% FBS. After 16 to 24 hours, cells on the membrane were fixed with 4% paraformaldehyde for 30 minutes and stained with trypan blue solution for 15 minutes at room temperature. Then the cells were imaged under a microscope.

#### Luciferase reporter assay.

Luciferase reporter assay was performed as described previously (60). Briefly, the promoter region of RPL14 containing the rs2276868-[C] or -[T] was cloned into a luciferase reporter pGL3-basic plasmid. Afterwards, 293T cells were cotransfected with 200 ng of pGL3-RPL14-C or -T constructs and 10 ng of pRL (Renilla luciferase) plasmid together with respective siRNAs or overexpression plasmids. After 48 hours, cells were harvested to measure the luciferase activity through a Dual-Luciferase Assay Kit (Promega) according to the manufacturer’s instructions. Specific cloning primers are listed in Supplemental Table 20.

#### Immunofluorescence staining and IHC.

Paraffin slides of xenograft tumors were deparaffinized with xylene and rehydrated through a gradient ethanol series. After microwave antigen retrieval in sodium citrate solution (pH 6), slides were incubated in 0.3% hydrogen peroxide for 15 minutes at room temperature to quench the endogenous peroxidase activity. Afterwards, the samples were incubated with the primary anti–Ki-67 or anti-CD34 antibodies (Supplemental Table 21) overnight at 4 °C. The next day, for immunofluorescence staining (IF), Alexa Fluor–conjugated secondary antibody (Thermo Fisher Scientific) was used for 1  hour at room temperature, followed by counterstaining with antifade mounting medium and DAPI for 10 minutes. Images were then captured with a confocal laser scanning microscope (Carl Zeiss, Microscope 880). For IHC, after exposure to secondary antibodies at room temperature for 1 hour, chromogenic detection was performed using 0.05% 3,30-diaminobenzidine (Dako). Hematoxylin was applied for counterstaining. Two independent pathologists from SYSUCC evaluated staining intensity. Afterwards, IHC scores were then calculated, combining staining intensity and the proportion of stained cells.

#### Manipulating gene expression using siRNA and lentivirus.

For transient knockdown of target genes, siRNAs specific against RPL14 or NKRF were transfected into cells using Lipofectamine RNAiMAX (Invitrogen) according to the manufacturer’s protocol. After 48–72 hours, cells were harvested to detect the knockdown efficiency of target genes through quantitative reverse-transcription PCR (RT-qPCR) as described below. Full-length cDNA of NKRF was cloned into the pcDNA3.1 vectors. RPL14, SELE-WT, and MUT were cloned into the pCDH-puro lentiviral vectors. For lentivirus preparation, 293T cells were transfected with the above lentivirus plasmids or control vector using Lipofectamine 2000 (Invitrogen) according to the manufacturer’s protocols. Afterwards, the supernatant media containing lentivirus were collected 2 times at 24 and 48 hours after transfection to infect NPC cell lines, following selection with puromycin (2 μg/mL) for at least 1 week. siRNAs or primers are listed in Supplemental Table 20.

#### RNA isolation, RT-qPCR, and ChIP PCR.

Total RNA was isolated using Trizol reagent (Invitrogen) following the manufacturer’s protocols. After determination of RNA concentration using a Nanodrop Spectrophotometer (Thermo Fisher Scientific), cDNA was synthesized using oligo (dT) primers and M-MLV Reverse Transcriptase (Promega) according to the manufacturer’s protocols. RT-qPCR was performed with the SYBR Premix Ex Taq Kit (Takara) on a LightCycler 96 (Bio-Rad). GAPDH or 18S RNA was used as an internal control. The primer sequences are shown in Supplemental Table 20. For ChIP assay, S26 cells transfected with Flag-NKRF plasmids or control vectors were grown in complete DMEM media to 80%–90% confluency. The media were removed and replaced with media containing 1% formaldehyde and crosslinked for 10 minutes at 37°C, followed by the ChIP assay with the SimpleChIP Plus Enzymatic Chromatin IP Kit (Magnetic Beads; CST) according to the manufacturer’s instructions. Afterwards, PCR or qPCR was performed with specific detection primers following the standard procedures (Supplemental Table 20).

#### EBV preparation and infection.

AKATA cells carrying EBV recombinant viruses were treated with 0.75 (v/v) of goat anti-human immunoglobulin G IgG (H0111-6, Tianfun Xinqu Zhenglong Biochem) for 6 hours at 37°C to induce the viral productive cycle. Virus supernatants were harvested 3 days after induction, filtered through a 0.45 μm filter, and centrifuged at 20,000*g* for 3 hours. Afterwards, virus concentrate was added into NPC cells and centrifuged at 2,000*g* in 37°C for 1 hour. Cell supernatants were removed and replaced with normal media 6 to 8 hours after infection. The infection efficiency was measured using flow cytometry analysis of GFP-positive cells.

#### Western blotting.

NPC cells were lysed in ice-cold cell lysis buffer (CST) containing 1× protease inhibitors (Beyotime). Cell lysate was prepared and subjected to Western blotting (detailed in Supplemental Note 10). Primary antibodies are commercially available and listed in Supplemental Table 21.

#### In vivo tumor xenograft.

Six-week-old male BALB/c nude mice (Beijing Vital River Laboratory Animal Technology) were grown in a specific pathogen–free (SPF) environment. For subcutaneous xenograft assay, 5 × 10^6^ CNE2-EBV cells or 2 × 10^6^ S26 cells coculturing with HUVEC cells (S26: HUVEC = 10:1) were suspended in 100 μL ice-cold PBS mixed with Matrigel (0.20 v/v, Corning Incorporated) and subcutaneously injected into the 2 flanks of the mice. Macroscopic observation and tumor volume measurement using a caliper were performed every 3 days. After 15 days, all mice were sacrificed, and tumor tissues were carefully dissected and weighed. Tumor volume was calculated with the following formula: tumor volume (mm^3^) = length (mm) × (width (mm)) ^2^/2. For tumor lymphatic metastasis assay, 1 × 10^6^ S26 cells coculturing with HUVEC cells (S26: HUVEC = 10:1) were injected into the foot pads of the node mice. After 1 month, the lymph nodes were harvested, the volume was calculated with the following formula: lymph node volume (mm^3^) = length (mm) × (width (mm)) ^2^ /2, and lymph nodes were paraffin embedded for H&E stains.

#### Tube formation assays.

For tube-formation assays, HUVEC cells stably expressing SELE-WT, the S149R mutant, or control vectors were resuspended in the ECM medium. Then the cells were seeded into 96-well plates precoated with growth factor–reduced Matrigel (Corning Inc. and incubated at 37°C for 5 hours. The formation of capillary-like structures was subsequently observed using a microscope. Quantitative analysis was conducted by counting the number of tube networks formed across the entire field, serving as an indicator of in vitro angiogenesis capability.

### Statistics

Statistical analyses were conducted using R, version 4, or Prism 8 (GraphPad). Association tests between genotypes and phenotype were performed using linear or logistic-based Wald test where appropriate. Continuous variables were compared using Student’s *t* test (2-tailed), Mann-Whitney *U* test (2-tailed), or ANOVA (1-way) where appropriate. Multiple testing correction was applied using Bonferroni’s, FDR, Šidák’s, or Dunnett’s methods where applicable. Unless otherwise specified, an adjusted *P* value < 0.05 was considered statistically significant.

### Study approval

The study was approved by the Sun Yat-sen University Cancer Center Ethics Committee in Guangzhou, China (reference no. SL-B2021-032-03), the SingHealth Institutional Review Board in SG (IRB protocol no. 2019/2177), and the IRB of the University of Hong Kong in Hong Kong, China. Informed consent was obtained from all participants. For animal studies in vivo, all experiments were performed in strict accordance with the instructions approved by the Institutional Animal Care and Use Committee of Sun Yat-sen University.

### Data availability

The genotype data generated by WES and Cap-seq have been deposited in the Genome Variation Map repository (https://ngdc.cncb.ac.cn/gvm/) of the National Genomics Data Center (NGDC) under controlled access due to data privacy laws related to patient consent for data sharing with accession number GVM000580. The NPC bulk RNA-Seq data are deposited at the OMIX repository (https://ngdc.cncb.ac.cn/omix/) of the NGDC (OMIX004586) and the NCBI’s Gene Expression Omnibus database (GEO GSE102349). The NPC scRNA-Seq data are available in the GEO database (GEO GSE162025, GSE150825, GSE150430) and Genome Sequence Archive (GSA) database under accession code HRA000087. Key data were deposited in the Research Data Deposit public platform (RDD: 2411010001, http://www.researchdata.org.cn). Values for all data points in graphs are reported in the Supporting Data Values file.

## Author contributions

JXB and YXZ designed the study. JXB, MLKC, MLL, and MJ procured financial support. MJ, FL, LX, CCK, ZL, MLL, JMYK, MLKC, EHWO, YMG, JRC, SH, SQL, XXC, XY, BW, YHZ, AYX, PPW, QYC, LQT, WHJ, and HQM recruited samples and prepared and collected the data. YZ, JXB, CLL, GWL, XB, YL, SH, ZHR, YLC, CCK, JJL, MLKC, JMYK, EHWO, JDM, DXL, and STL analyzed and interpreted data.CLL, JXJ, WXY, and YQZ performed functional experiments. YZ, CLL, and JXB wrote the paper (original draft); all authors approved the final report.

## Supplementary Material

Supplemental data

Unedited blot and gel images

Supplemental tables 1-21

Supporting data values

## Figures and Tables

**Table 2:**
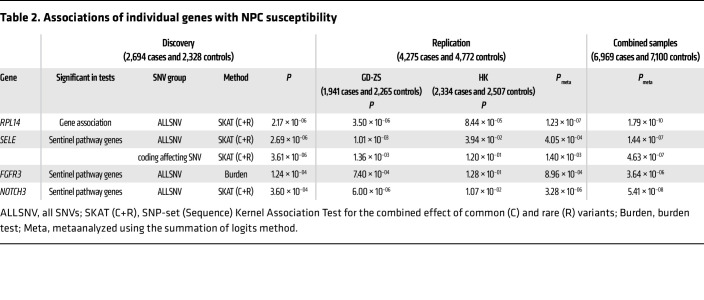
Associations of individual genes with NPC susceptibility

**Table 1:**
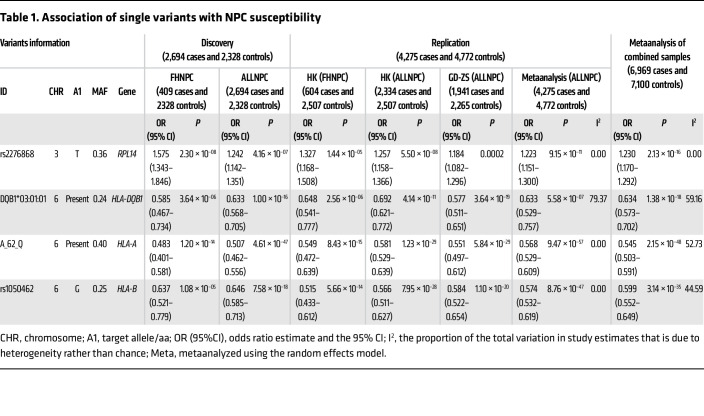
Association of single variants with NPC susceptibility

## Version 1 01/02/2025

Electronic publication
