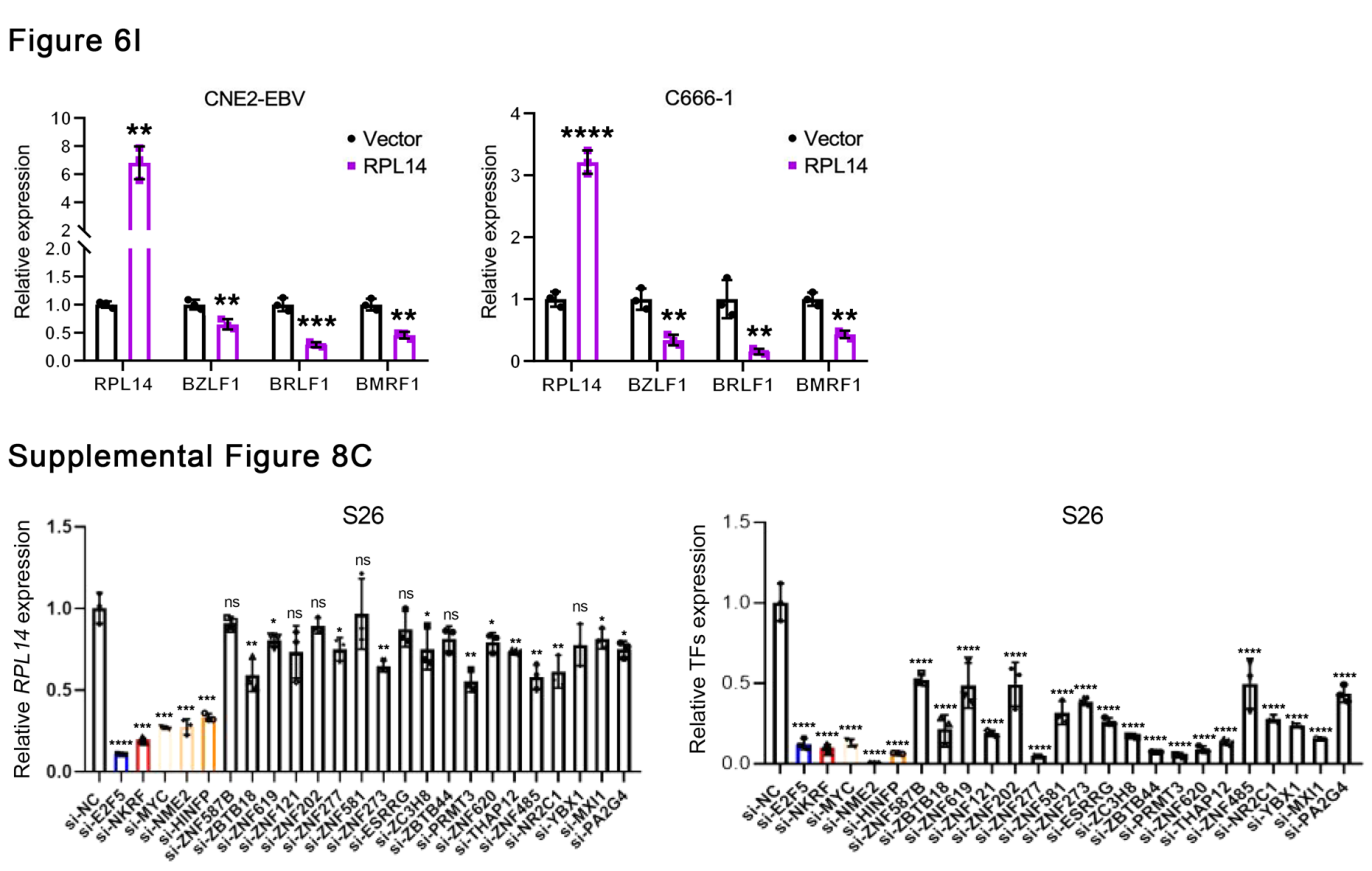# Corrigendum to Whole-exome sequencing association study reveals genetic effects on tumor microenvironment components in nasopharyngeal carcinoma

**DOI:** 10.1172/JCI209768

**Published:** 2026-08-03

**Authors:** Yanni Zeng, Chun-Ling Luo, Guo-Wang Lin, Fugui Li, Xiaomeng Bai, Josephine Mun-Yee Ko, Lei Xiong, Yang Liu, Shuai He, Jia-Xin Jiang, Wen-Xin Yan, Enya Hui Wen Ong, Zheng Li, Ya-Qing Zhou, Yun-He Zhou, An-Yi Xu, Shu-Qiang Liu, Yun-Miao Guo, Jie-Rong Chen, Xi-Xi Cheng, Yu-Lu Cao, Xia Yu, Biaohua Wu, Pan-Pan Wei, Zhao-Hui Ruan, Qiu-Yan Chen, Lin-Quan Tang, James D. McKay, Wei-Hua Jia, Hai-Qiang Mai, Soon Thye Lim, Jian-Jun Liu, Dong-Xin Lin, Chiea Chuen Khor, Melvin Lee Kiang Chua, Mingfang Ji, Maria Li Lung, Yi-Xin Zeng, Jin-Xin Bei

Original citation: *J Clin Invest*. 2025;135(1):e182768. https://doi.org/10.1172/JCI182768

Citation for this corrigendum: *J Clin Invest*. 2026;136(15):e209768. https://doi.org/10.1172/JCI209768

After publication of this article, the authors became aware of the following errors: in Figure 6I, there was a typo in the gene name *BRLF1*; and in Supporting Data Values only, the values for the CNE2-EBV cell line were duplicated as C666-1 values. In the left panel of Supplemental Figure 8C, the values for ZC3H8 and ZBTB44 were incorrectly shown as identical, corresponding to data inadvertently duplicated in Supporting Data Values.

The correct figures are shown. The HTML and PDF versions, including the supplemental material and Supporting Data Values, have been updated.

The author regrets the errors.

## Figures and Tables

**Figure 6I and Supplemental Figure 8C:**