# Supplementary material for: Whole-exome sequencing association study reveals genetic effects on tumor microenvironment components in nasopharyngeal carcinoma
Source: J Clin Invest. 2025 Jan 2;135(1):e182768. doi: 10.1172/JCI182768 (PMC11684818; doi:10.1172/JCI182768)
Supplement: Supplemental data [file jci-135-182768-s144.pdf]

Supplemental Notes

Supplemental Note 1. sample and data collections

Discovery samples

A total of 3,039 NPC patients of Chinese Han ancestry was recruited from Guangzhou in Guangdong province of China (GD-SYSUCC; n= 2,196) by SYSUCC and from Singapore (SG; n=843) by the National Cancer Center (Singapore, collected from 1987-2018). A total of 2,692 healthy individuals of Chinese Han ancestry was recruited as controls from local community in Guangdong (GD-SYSUCC; n=1,355) and from Singapore (SG; n =1,337) by Genome Institute of Singapore (Singapore, details have been described previously (1)). The Chinese Han ancestry was self-declared and confirmed by PCA analysis (supplemental Figure S2). All NPC cases were histologically diagnosed. All healthy controls declared free of cancer through questionnaires. A total of 709 samples were excluded due to relatedness or genetic outlier (n = 581), calling quality or heterozygosity deviation (n = 128), remaining 5022 samples (2,694 cases and 2,328 healthy controls) for downstream analyses.

Replication samples

NPC cases and controls in the GD-ZS cohort were collected by Zhongshan City People's Hospital. NPC cases and controls in the HK cohort were collected by the Tissue Bank established under the Area of Excellence (AoE) program from five Hong Kong public hospitals including Queen Mary Hospital (QMH), Queen Elizabeth Hospital (QEH), Tuen Mun Hospital (TMH), Pamela Youde Nethersole Eastern Hospital (PYNEH), and Princess Margaret Hospital (PMH) from 2010 to 2017, details have been described previously(2). NPC cases were confirmed by histopathology and

enrolled with routine staging procedures according to the American Joint Committee on Cancer (AJCC) TNM system, physical examination, and imaging tests. The control population was recruited from the Red Cross and hospital cancer-free individuals from QMH, QEH, TMH, PYNEH, and PMH. The initial collection of replication samples included 4,321 NPC cases and 4,808 controls, of which 82 were excluded for calling quality or gender mismatch, remaining 4,275 cases and 4,772 controls in downstream analyses.

scRNA-seq data of other cancers

Lung cancer tumor dataset (n_{sample}=19, n_{cell}=90,477) was downloaded from the GEO database (GSE131907, GSE139555)(3); the gastric cancer tumor dataset (n_{sample}=9, n_{cell}=28,299)(4); the colorectal cancer tumor dataset (n_{sample}=37, n_{cell}=70,015) downloaded from the GEO database (GSE132465, GSE144735 and GSE139555).

Supplemental Note 2. Library construction, quality control, variant calling (including HLA genotyping) and variant annotation for WES data

Library construction

Library was constructed using one of the three products: Agilent SureSelect Human All Exon V6+UTR kit, Agilent SureSelect Human All Exon V5+UTR kit, and Roche SeqCap EZ Exome + UTR Target Enrichment Kit.

Baseline quality control and variant calling

A custom optimized version of Sentieon (5) was applied to preprocess sequencings reads, align reads to human genome using GRCh37 (hg19) assembly as reference, and conduct single nucleotide variant (SNV) calling using algorithms following the GATK best-practice pipeline (6, 7). After GATK's VQSR quality control, hard filtering was applied to remove the variants with 1) Read depth (DP) <10, 2) Allelic Balance (AB) > 0.8 or < 0.2 for heterozygous genotype, or 3) Genotype quality (GQ) < 30. For rare variants (minor allele frequency or MAF < 0.01), we applied additional stringent quality control based on per-read mutation rate, allelic balance, and strand bias (defined as proportion of allele called by reads from one strand over that by any of the two strands). We used the high confident SNP set derived from the 1000 genome phase 1 as golden set to establish parameter threshold range where high liability variants can be called. This led to the following filtering for rare variants: 1) refining alignment by removing any read with a mismatch distance against the reference larger than 8 bp (any indel event counts for 1 bp) and re-apply hard filtering as described above; 2) setting variant-sample elements with strand bias > 0.95 or < 0.05 for heterozygous genotype, or >0.97 or <0.03 for homozygous genotype to missing; 3) setting variant-sample elements with allelic Balance (AB) > 0.75 or < 0.22 for heterozygous genotype , or with allelic Balance (AB) < 0.95 and > 0.04 for homozygous genotype to missing.

HLA genotyping

High resolution WES-based HLA typing was performed on the bam files using HLAScan (v2.1.3) with default parameters and implemented HLA database(8). PCR amplification HLA typing was also performed on 135 WES samples to cross-validate the WES-based typing procedures. The concordance of the HLA genotypes was high (> 94%) at four-digit resolution level for most HLA genes, except for HLA-DQA1 and -DPB1 with a relatively lower concordance being observed (>

80%). The HLA amino acid (AA) polymorphism was obtained using HLA AA alignments in IPD-IMGT database(9). The genotypes of HLA SNPs were inferred by the SNP2HLA software(10).

Additional quality control for association test

For both common and rare variants, we further removed variants that are 1) with a missing rate >= 0.02, 2) with a Hardy-Weinberg equilibrium P value <= 1 x 10^{-6} in healthy controls, 3) with a significant differential missing rate between case vs controls (Bonferroni corrected), or 4) variants in super duplicates or low complexity regions. Samples were removed if they are 1) with a missing rate > 0.05, 2) with autosomal heterozygosity deviation outside 3 standard deviations of the mean, or 3) genetic outliers through eye check or with any the of top four principal component value located outside 6 standard deviations of the mean. These filtering steps were performed using PLINK(11). We also removed one of each related pair (remained sample t<0.05) using GCTA(12).

Variant annotation

ANNOVAR was used to annotate SNV with following categories(13):

1) SNPs in dbSNP150 database;

2) Variants in population genetics database: 1000 genome phase 3, exome and genome subset of the Genome Aggregation Database (EXAC, gnomAD);

3) within genes in NCBI RefSeq Gene database;

4) within genes of pathways in curated canonical pathways, GO Ontology items, and oncogenic signature gene sets as downloaded from Molecular Signatures Database v6.2(14);

5) variants that have potential functional consequences as they ranked at top 5% of the rank score of CADD (Combined Annotation Dependent Depletion), or DANN (deleterious annotation of genetic variants using neural networks) or fitCons (fitness consequence score) (15-17);
6) ‘pathogenic’ or ‘likely pathogenic’ variants as annotated in InterVar database (clinical interpretation of missense variants)(18).

Supplemental Note 3. Capture sequencing

DNA library preparation was performed as described in the NadPrep DNA Library Preparation Kit (#1002222; Nanodigmbio, Nanjing, China) for the MGI sequencing platform on the MGISP-960 automated workstation (MGI Tech Co., Ltd., Shenzhen, China), in accordance with the manufacturers’ instructions. Hybridization capture-based target enrichment was performed as described in the NadPrep Hybrid Capture Reagents kit (#1005101; Nanodigmbio) on the MGISP-100 automated workstation (MGI Tech Co., Ltd., Shenzhen, China), in accordance with the manufacturers’ instructions. The library distribution was analyzed by Qsep100 (BiOptic Inc, New Taipei City, China) and quantified by Qubit dsDNA HS Assay Kit (Invitrogen, Termo Fisher Scientifc Inc, Waltham, USA). Final libraries were sequenced on MGI DNBSEQ-T7 (MGI Tech Co., Ltd., Shenzhen, China) using a pair-end of 150 bp. For variant calling, the same analytic pipeline as above WES data was applied.

Supplemental Note 4. SNV-set-based association analysis

The following four algorithms were applied for two types of SNVs, all SNVs (SNV type 1 or ALL_SNV) or only coding-affecting SNVs (SNV type 2 or CODING_SNV):

1) The original SNP-set (Sequence) Kernel Association Test (SKAT)(19);

2) The original Burden test(20);

3) SKAT for the combined effect of common and rare variants using the sum test as described previously(21);

4) Burden test for the combined effect of common and rare variants using the sum test as described previously(21).

Supplemental Note 5. Gene expression quantification, cell type determination and inference of malignant status of epithelial cells in scRNA-seq data

The quantification of gene expression and determination of cell types for NPC datasets were performed separately. Specifically, we first used DoubletFinder to detect doublets (22) by inspecting abnormal clusters that simultaneously express two or more cell type markers. For each sample, doublets were removed with the expected doublet rate of 0.05. Cells with $n_{Feature\_RNA} > 500$, $n_{Count\_RNA} > 1,000$, and percent.mt < 25% were retained for downstream analyses. Expression matrix of all cells were converted to a Seurat object and managed by Seurat's pipeline(23): normalization with NormalizeData and ScaleData, batch effect correction with Harmony(24), dimensionality reduction with RunUMAP, clustering with FindNeighbors (k.param = 30), and FindClusters. Clusters were annotated by classic markers (brackets) as following: NK cells (KLRF1), T cells (CD3D), Plasma cells (MZB1), B cells (MS4A1), Myeloid cells (AIF1), Cancer Associated Fibroblasts (COL1A1), Endothelial cells (VWF), and Epithelial cells (EPCAM). Malignant cells have substantially increased copy number variation (CNV), such as large-scale gain or deletions of chromatin. Therefore, we used inferCNV to identify malignant epithelial cell

clusters (58). Comparing with control normal epithelial cells downloaded from GEO database (GSE121600), all epithelial cell clusters identified in NPC scRNA-seq data in our study were considered malignant.

Lung, gastric, colorectal cancer tissue datasets were merged and analyzed. We first used Scrublet (25) to remove estimated doublets and SoupX (26) to remove cell-free mRNA contamination. Cells with $n_{Feature\_RNA} > 500$, $n_{Count\_RNA} > 1000$, and percent.mt < 25% through quality control were kept for downstream analysis. NormalizeData and ScaleData in Seurat was used to normalize data (27); FindVariableFeatures with $n_{feature} = 3000$ was used to select hypervariable genes. We used Harmony to correct for batch effect caused by different patients (24), and used RunUMAP with dims = 1:30 and FindClusters with resolution = 0.3 for dimension reduction and clustering. The clusters were annotated by classic markers of major cell types.

Supplemental Note 6. GREML-LDMS

In brief, when calculating the total WES SNV-heritability and the proportion explained by variants from HLA and non-HLA regions, SNVs were stratified into four groups based on MAF and genomic regions: MAF ≥ 0.01 in HLA region, MAF<0.01 in HLA region, MAF ≥ 0.01 in non-HLA region, and MAF < 0.01 in non-HLA region. When further decomposing the WES SNV-heritability in non-HLA region, SNVs were stratified into 28 groups based on LD scores in four bins based on quartiles and MAF in seven bins. LD score was calculated as the sum of R^2 with variants in a 200kb window and the MAF bins are: 0.0005 < MAF <= 0.001 ,0.001 < MAF <=0.01, 0.01 < MAF <=0.1, 0.1 < MAF <= 0.2, 0.2 < MAF <=0.3, 0.3 < MAF <=0.4, and 0.4 < MAF <=0.5. For each SNV group, genomic relationship matrix (GRM) was created. In each model (the four components and 28 components ones), GRMs from all groups were then simultaneously fitted

in the GREML model for NPC, where the total NPC variation explained by all genetic components and the variation explained by individual genetic component as represented by each GRM can by estimated.

Supplemental Note 7. Joint model for comparative analyses of genetic effect of different sources

In discovery samples, a subset (1,382 cases and 912 controls) of the GD-SYSUCC cohort simultaneously have imputed GWAS array(28) and WES data available, allowing the comparisons of the relative disease risks conferred by the common variant rs2276868 in *RPL14* and the rare variant *rs5361* in SELE with the polygenic risk from the HLA and other known GWAS loci. In replication samples, the cap-seq designed to target both published GWAS loci and the candidate loci identified by the present WES study also enabled this comparison. In brief, for each cohort we applied a joint regression model that simultaneously accounts for genetic effects from different variants: the effects from *RPL14* and *SELE* were presented by the genotypes of rs2276868 (*RPL14*) and rs5361(*SELE*), respectively; the combined genetic effects from published non-HLA GWAS loci and that from HLA loci were represented as two polygenic risk scores (one for non-HLA GWAS loci and one for HLA loci) constructed using odds ratios estimated from original publication or our single-variant analysis using discovery cohorts (Supplemental Table S18).

Supplemental Note 8. rcPRS and gPRS

Construction of rcPRS

SNVs were used to create rcPRS include:

1) In WES data, significant variants in single-variant-based association test (including HLA variants identified in conditional analysis);
2) In WES data, leading variants in gene-based test if they:
   a. were in genes significant in gene-based association test, and
   b. reached $P<1\times10^{-4}$ in single-SNV-based association test.
3) In WES data, leading variants in pathway-based test as they:
   a. were in a gene of significant pathways and that gene has a P<0.01 in gene-based test; and
   b. reached $P<1\times10^{-4}$ in single-SNV-based association test.
4) Published GWAS loci (28, 29). Only non-HLA GWAS loci was extracted from this source as majority of HLA SNPs identified in published GWAS were in non-coding regions, which are beyond the regions captured by WES and that signals from HLA regions have already been included in source 1.

To avoid duplication due to overlapped genes among pathways, each variant can be used only once. Clumping was performed so that in each cluster of LD partners with $R^2 \geq 0.1$ (within 250kb extended windows) only one most significant variant could be remained. PRS was created as the sum of weighted genetic risk from all variants. For each variant, the weighted genetic risk was calculated as the product of allele dosage and the coefficient of variant effect estimated in single-variant-based test. For variants from published GWAS, the coefficient was extracted from original publications (28-30).

Construction of gPRS

For subset samples in GD-SYSUCC cohort with BeadChips array data available, we created gPRS, In brief, SNP array data was obtained from a previous study (29). To prepare the data for imputation analysis, we first used Liftover to convert the SNP array data from hg18 to hg19 encoding strand. Next, we performed quality control at the SNP level, removing SNPs with a MAF less than 0.01, genotype missing rate greater than 0.05, and those that failed the Hardy-Weinberg equilibrium test (HWE) with a *P*-value less than 10^{-5}. A total of 463,270 SNPs passed these quality control criteria and were kept for further analysis. For the quality-controlled genotype data, we used SHAPEIT2 with default parameters to estimate haplotypes of the study samples (31). We then used IMPUTE2 with default parameters and the haplotype data from the 1000 Genomes Project to impute the missing genotype of each individual's haplotype (32). After imputation, we removed potentially low-quality SNPs with an INFO value less than 0.8 and set loci with genotype probability (GP) values less than 0.9 to missing. Ultimately, we retained 6,095,964 SNPs for analysis. Construction of gPRS follows the instruction of a previous study (33). To note, for three SNPs (rs2106123, rs6475604, rs9507124) not genotyped and not imputed in GD-SYSUCC, we used three surrogate SNPs in complete LD (R^2=1 in Chinese Southern Han population in 1000 genome phase 3 dataset) with them (rs6774494 for rs2106123, rs1412829 for rs6475604, and rs9510790 for rs9507124) in the model.

Supplemental Note 9. Construction of composite scores for pathway and EBV activity

Using on AddModuleScore, the pathway score is obtained by subtracting the average expression of the corresponding control gene set from the average expression of the target gene set. For samples with scRNA-seq, we took each patient as a datapoint, meaning that the average expression of a given gene across all cells of the cell type of interest from the patient was treated as the

expression of that gene for the patient. Composite scores for EBV activity were also constructed. In brief, we downloaded the EBV gene list from the EBV reference sequence (Akata; https://github.com/flemingtonlab/public/tree/master/annotation) and mapped the scRNA-seq data of EBV-positive malignant epithelial cells onto them. We then used the annotated EBV gene data to construct an EBV-composite score for each NPC patient with scRNA-seq data derived from EBV-positive epithelial cells available, following the same steps as described above.

Supplemental Note 10. Western blotting

Cell lysate was cleared by centrifugation at 14,000 ×g for 10 min at 4°C and boiled in 1× LDS loading buffer at 100°C for 10 min. Proteins were separated by SDS-PAGE and transferred to 0.2 μm polyvinylidene difluoride (PVDF) membrane (Merck Millipore, USA). Membranes were blocked with 5% bovine serum albumin (BSA; Sangon Biotech, China) in tris-buffered saline and Tween 20 (TBST) at room temperature for 1h. Afterwards, the membranes were incubated with target-specific antibodies in blocking buffer at 4°C overnight. Next day, horseradish peroxidase–conjugated (HRP) secondary antibodies were used at room temperature for 1h. Proteins were then detected with Fdbio-Dura ECL kit (Fdbio science, China) and quantified by Bio-Rad ChemiDoc Touch (Hercules, USA).

References

1. Li Z, et al. Association of rare CYP39A1 variants with exfoliation syndrome involving the anterior chamber of the eye. 2021;325(8):753-64.
2. Ning L, et al. Nasopharyngeal carcinoma MHC region deep sequencing identifies HLA and novel non-HLA TRIM31 and TRIM 39 loci. 2020;3(1):759.
3. Qian J, et al. A pan-cancer blueprint of the heterogeneous tumor microenvironment revealed by single-cell profiling. 2020;30(9):745-62.

4. Zhang M, et al. Dissecting transcriptional heterogeneity in primary gastric adenocarcinoma by single cell RNA sequencing. 2021;70(3):464-75.
5. Aldana R, and Freed D. Data Processing and Germline Variant Calling with the Sentieon Pipeline. *Methods in molecular biology.* 2022;2493:1-19.
6. Kendig KI, et al. Sentieon DNASeq variant calling workflow demonstrates strong computational performance and accuracy. 2019;10.
7. DePristo MA, et al. A framework for variation discovery and genotyping using next-generation DNA sequencing data. 2011;43(5):491.
8. Ka S, et al. HLAscan: genotyping of the HLA region using next-generation sequencing data. 2017;18(1):1-11.
9. Robinson J, et al. Ipd-imgt/hla database. 2020;48(D1):D948-D55.
10. Jia X, et al. Imputing amino acid polymorphisms in human leukocyte antigens. 2013;8(6):e64683.
11. Purcell S, et al. PLINK: A tool set for whole-genome association and population-based linkage analyses. *American journal of human genetics.* 2007;81(3):559-75.
12. Yang J, et al. GCTA: a tool for genome-wide complex trait analysis. *American journal of human genetics.* 2011;88(1):76-82.
13. Wang K, et al. ANNOVAR: functional annotation of genetic variants from high-throughput sequencing data. *Nucleic acids research.* 2010;38(16):e164.
14. Subramanian A, et al. Gene set enrichment analysis: a knowledge-based approach for interpreting genome-wide expression profiles. *Proceedings of the National Academy of Sciences of the United States of America.* 2005;102(43):15545-50.
15. Quang D, et al. DANN: a deep learning approach for annotating the pathogenicity of genetic variants. *Bioinformatics.* 2015;31(5):761-3.
16. Rentzsch P, et al. CADD: predicting the deleteriousness of variants throughout the human genome. *Nucleic acids research.* 2019;47(D1):D886-D94.
17. Gulko B, et al. A method for calculating probabilities of fitness consequences for point mutations across the human genome. *Nature genetics.* 2015;47(3):276-83.
18. Li Q, and Wang K. InterVar: Clinical Interpretation of Genetic Variants by the 2015 ACMG-AMP Guidelines. *American journal of human genetics.* 2017;100(2):267-80.
19. Wu MC, et al. Rare-variant association testing for sequencing data with the sequence kernel association test. 2011;89(1):82-93.
20. Li B, and Leal SMJTAJoHG. Methods for detecting associations with rare variants for common diseases: application to analysis of sequence data. 2008;83(3):311-21.
21. Ionita-Laza I, et al. Sequence kernel association tests for the combined effect of rare and common variants. 2013;92(6):841-53.
22. McGinnis CS, et al. DoubletFinder: doublet detection in single-cell RNA sequencing data using artificial nearest neighbors. 2019;8(4):329-37. e4.
23. Hao Y, et al. Integrated analysis of multimodal single-cell data. 2021;184(13):3573-87. e29.
24. Korsunsky I, et al. Fast, sensitive and accurate integration of single-cell data with Harmony. 2019;16(12):1289-96.
25. Wolock SL, et al. Scrublet: computational identification of cell doublets in single-cell transcriptomic data. 2019;8(4):281-91. e9.
26. Young MD, and Behjati SJG. SoupX removes ambient RNA contamination from droplet-based single-cell RNA sequencing data. 2020;9(12):giaa151.

27. Hao Y, et al. Integrated analysis of multimodal single-cell data. *Cell.* 2021;184(13):3573-87 e29.
28. Cui Q, et al. An extended genome-wide association study identifies novel susceptibility loci for nasopharyngeal carcinoma. 2016;25(16):3626-34.
29. Bei JX, et al. A genome-wide association study of nasopharyngeal carcinoma identifies three new susceptibility loci. *Nature genetics.* 2010;42(7):599-603.
30. Bei J-X, et al. A GWAS Meta-analysis and Replication Study Identifies a Novel Locus within CLPTM1L/TERT Associated with Nasopharyngeal Carcinoma in Individuals of Chinese AncestryNPC GWAS Follow-up Identifies CLPTM1L/TERT Locus. 2016;25(1):188-92.
31. Delaneau O, et al. A linear complexity phasing method for thousands of genomes. 2012;9(2):179-81.
32. Howie BN, et al. A flexible and accurate genotype imputation method for the next generation of genome-wide association studies. 2009;5(6):e1000529.
33. He YQ, et al. A polygenic risk score for nasopharyngeal carcinoma shows potential for risk stratification and personalized screening. *Nature communications.* 2022;13(1):1966.

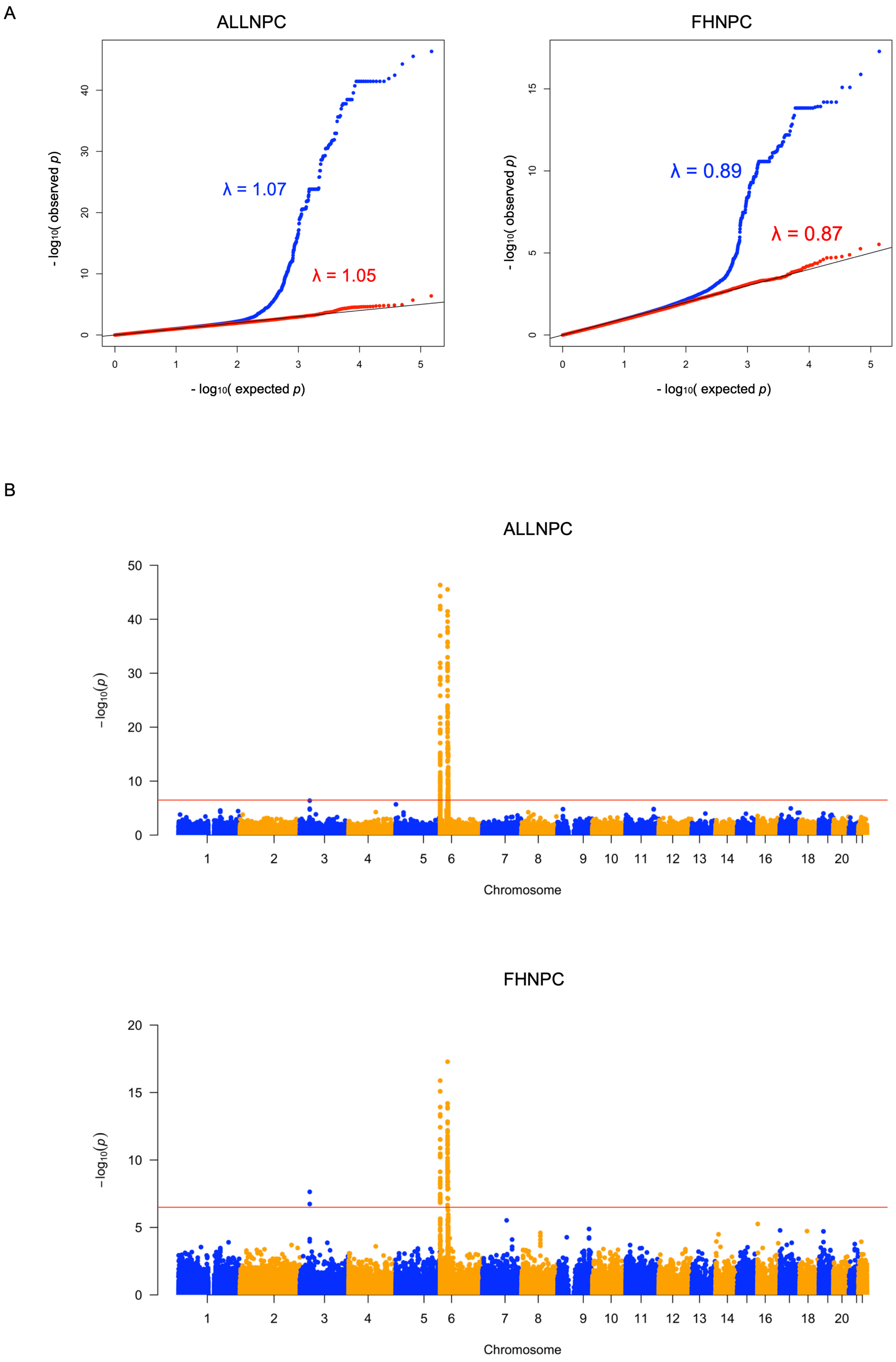

Figure S1. Quantile-Quantile and Manhattan plots of single-variant-based association test. ALLNPC: all NPC cases. FHNPC: NPC cases with a family history of NPC. (**A**). Quantile-Quantile plots. λ: Genomic Inflation Factor. Each dot represents one SNP, colored in Blue for all tested SNPs and Red for SNPs removing variants from MHC region (ALLNPC and FHNPC) and RPL14 locus (FHNPC). (**B**). Manhattan lots. Red line: significant threshold based on Bonferroni's method ($P < 3.2 \times 10^{-7}$).

A

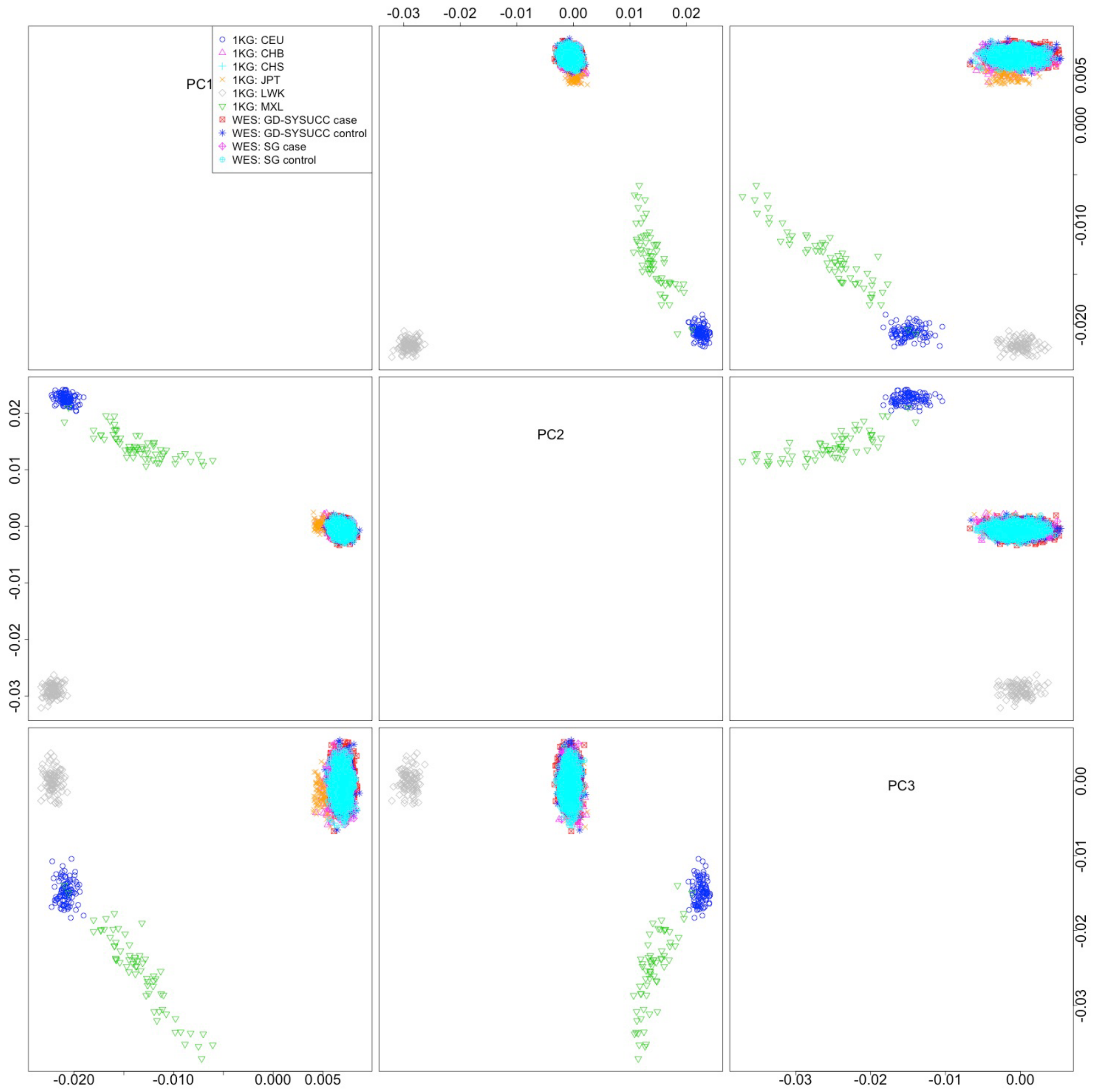

1KG: CEU
1KG: CHB
1KG: CHS
1KG: JPT
1KG: LWK
1KG: MXL
WES: GD-SYSUCC case
WES: GD-SYSUCC control
WES: SG case
WES: SG control
PC1
PC2
PC3
-0.03 -0.02 -0.01 0.00 0.01 0.02
0.005 0.000 -0.010 -0.020
0.02 0.01 0.00 -0.01 -0.02 -0.03
0.00 -0.01 -0.02 -0.03
-0.020 -0.010 0.000 0.005
-0.03 -0.02 -0.01 0.00

B

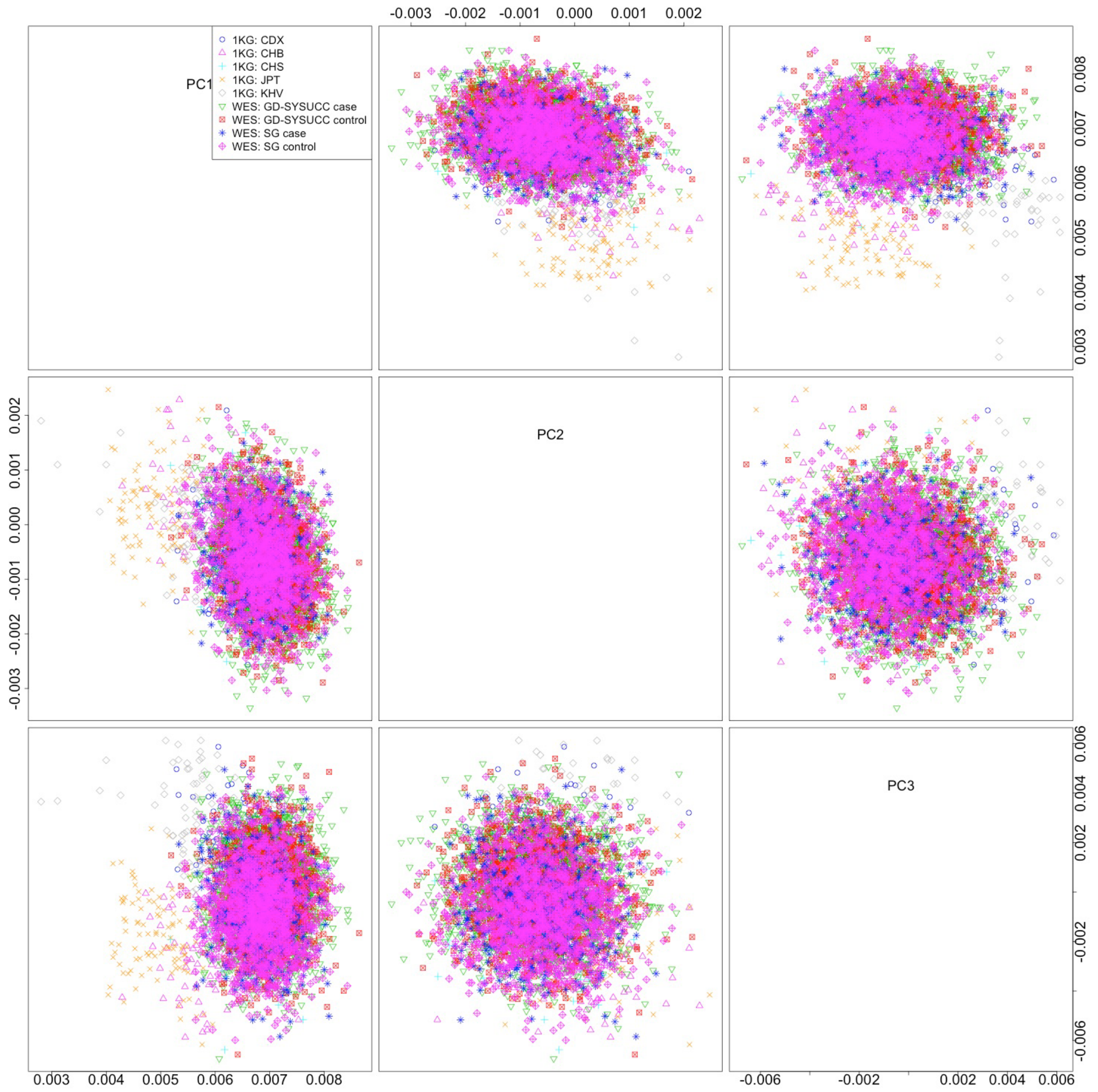
1KG: CDX
1KG: CHB
1KG: CHS
1KG: JPT
1KG: KHV
WES: GD-SYSUCC case
WES: GD-SYSUCC control
WES: SG case
WES: SG control
PC1
PC2
PC3
-0.003 -0.002 -0.001 0.000 0.001 0.002
0.003 0.004 0.005 0.006 0.007 0.008
-0.006 -0.002 0.002 0.004 0.006

c

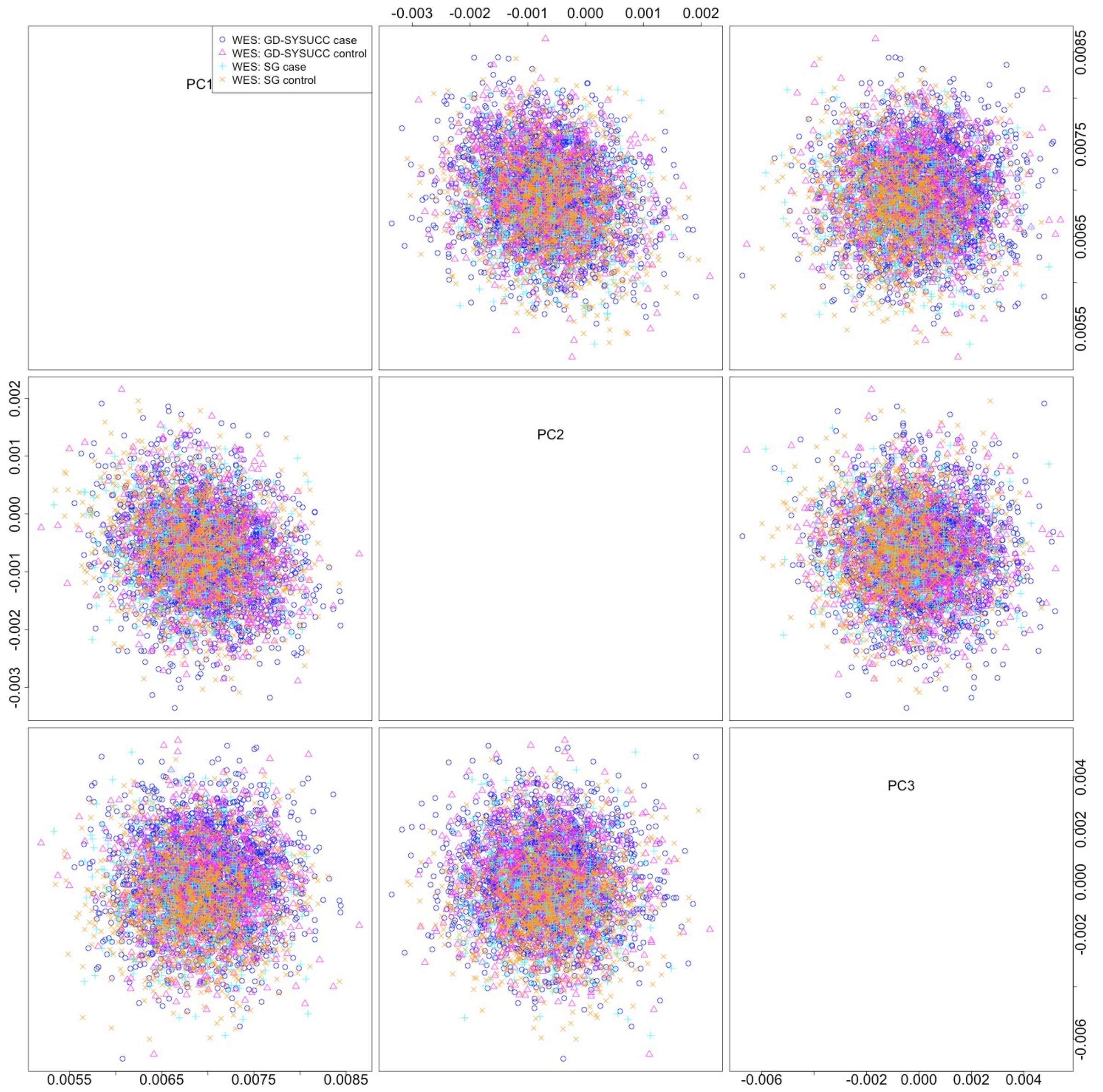

Figure S2. Plots of top three genetic principal components for the discovery samples and the referenced population samples in 1000 genome reference data (phase 3). Top, discovery WES samples and global populations samples in 1000 genome reference data; middle, discovery WES samples and East Asia populations samples in 1000 genome reference data; bottom, zoom-in view for discovery WES samples. WES: inhouse WES samples in discovery stage. 1KG: population samples in 1000 genome reference data (phase 3); CHB: Han Chinese in Beijing, China; CHS: Han Chinese South, China, China; JPT: Japanese in Tokyo, Japan; CEU: Utah Residents (CEPH) with Northern and Western European ancestry; LWK: Luhya in Webuye, Kenya; MXL: Mexican Ancestry from Los Angeles USA. PC: principal component.

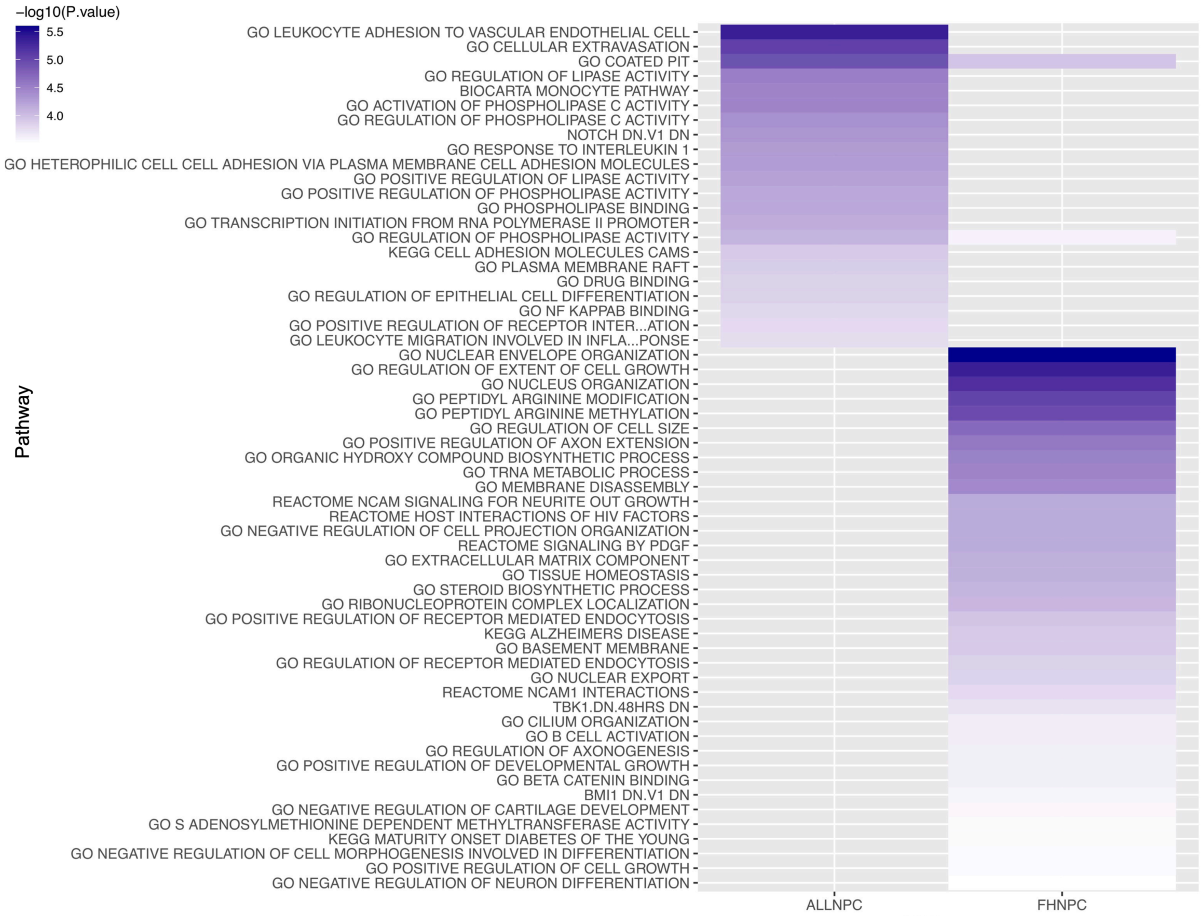

Figure S3. Significant pathways associated with NPC. Significant pathways associated with NPC. ALLNPC denotes all NPC samples in the discovery stage, whereas FHNPC represents NPC cases with a familial NPC history. Color bars from white to purple indicate minus log-transformed *P* values.

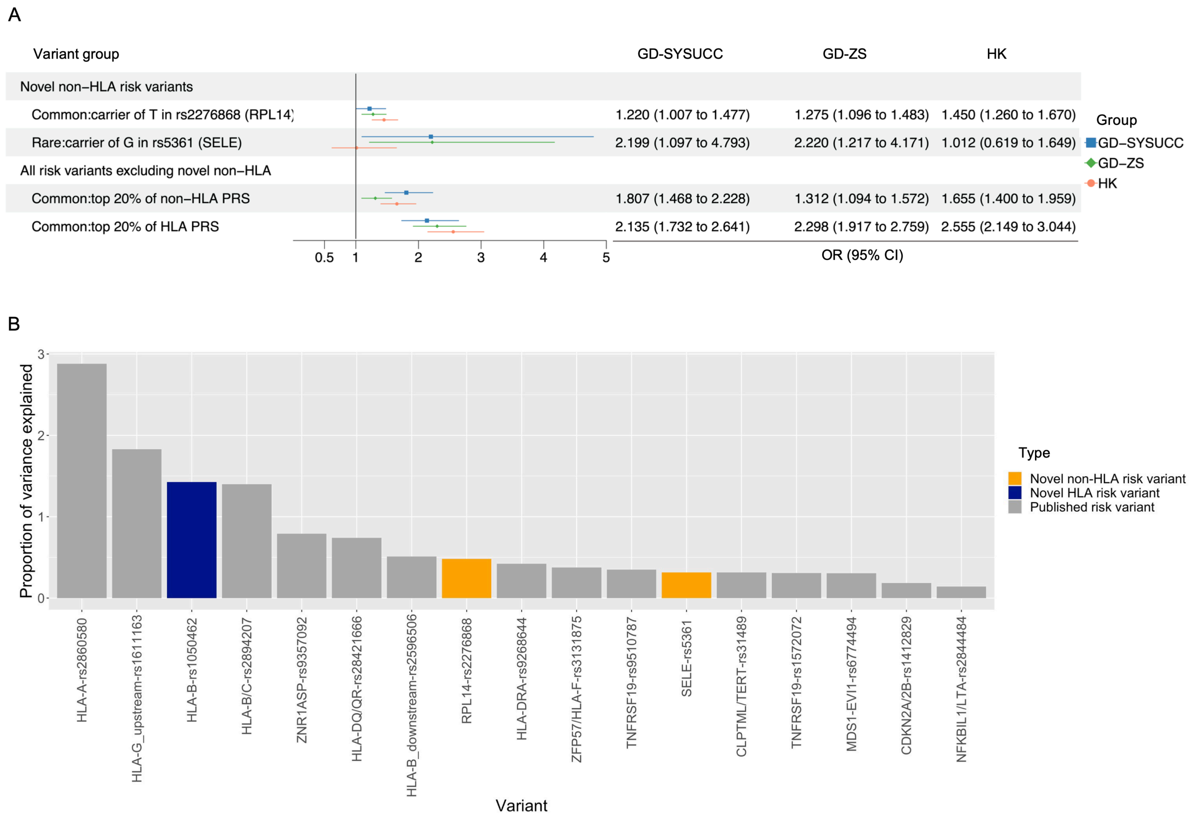

Figure S4. Dissecting genetic risk for NPC contributed by the identified and previously known loci. (**A**). Detailed odds ratio representation for NPC risk attributed from genetic effects of different variant categories in sample groups. (**B**). bar chart visualization of the disease risk broken down by variant types. Orange: novel NPC-associated variants from the non-HLA region, blue: novel NPC-associated variant within HLA region, grey: previously known NPC-associated variants through GWAS.

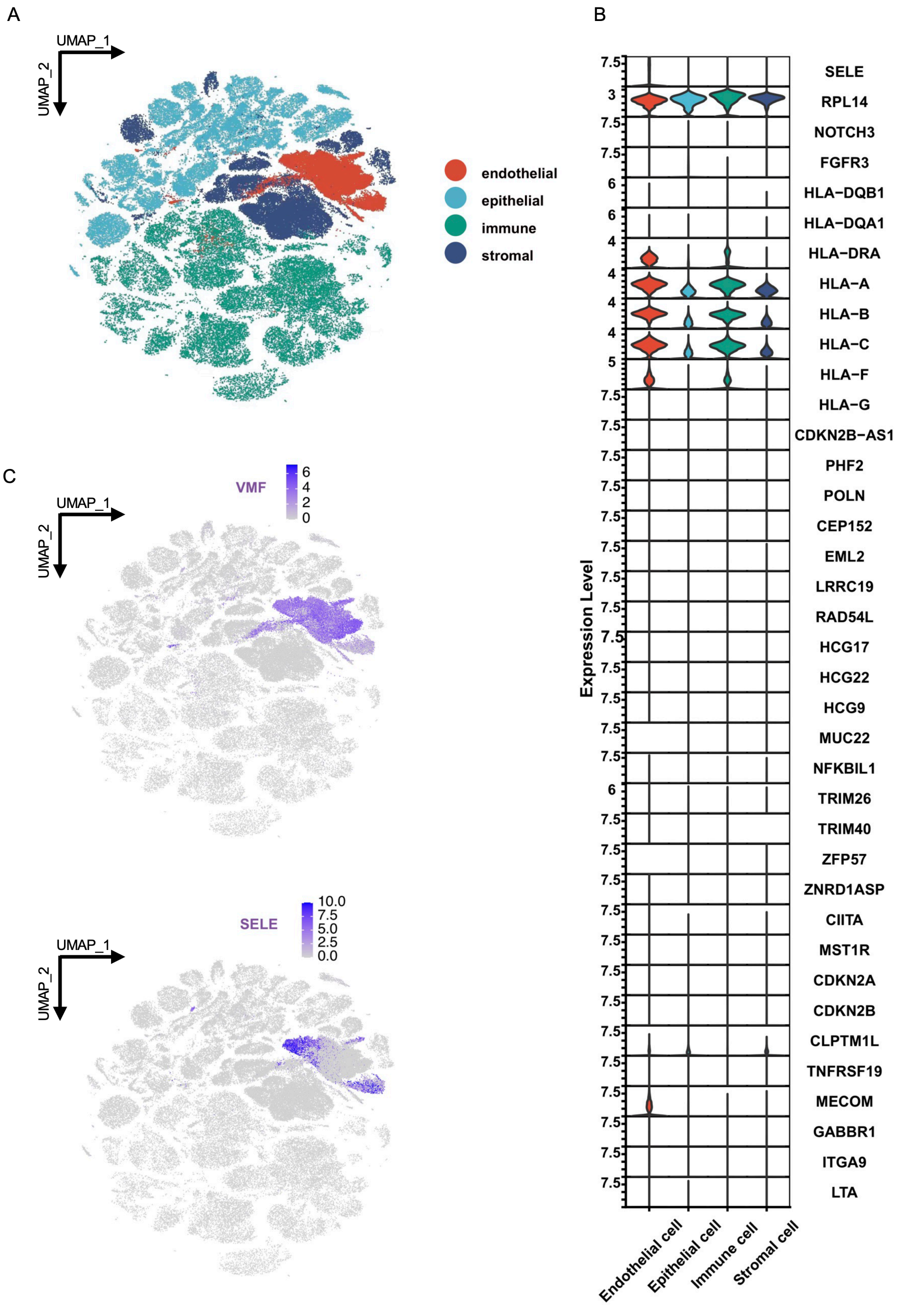

Figure S5. Expression patterns of novel and known NPC-associated genes across diverse cell types in non-tumor tissues. Single-cell transcriptomic analyses of 80,000 cells randomly sampled from data of non-tumor tissues of 15 individuals without cancer. (**A**). UMAP plot of 80,000 single cells grouped into four major cell clusters. (**B**). Violin plot of normalized expression of NPC-associated genes in major cell clusters. As the endothelial cell class has been showed separately, the stromal cell class only included the stromal cells not belonging to the endothelial group. (**C**). The expression of the maker gene (VWF) for endothelial cells alongside the novel NPC-associated gene *SELE*.

A

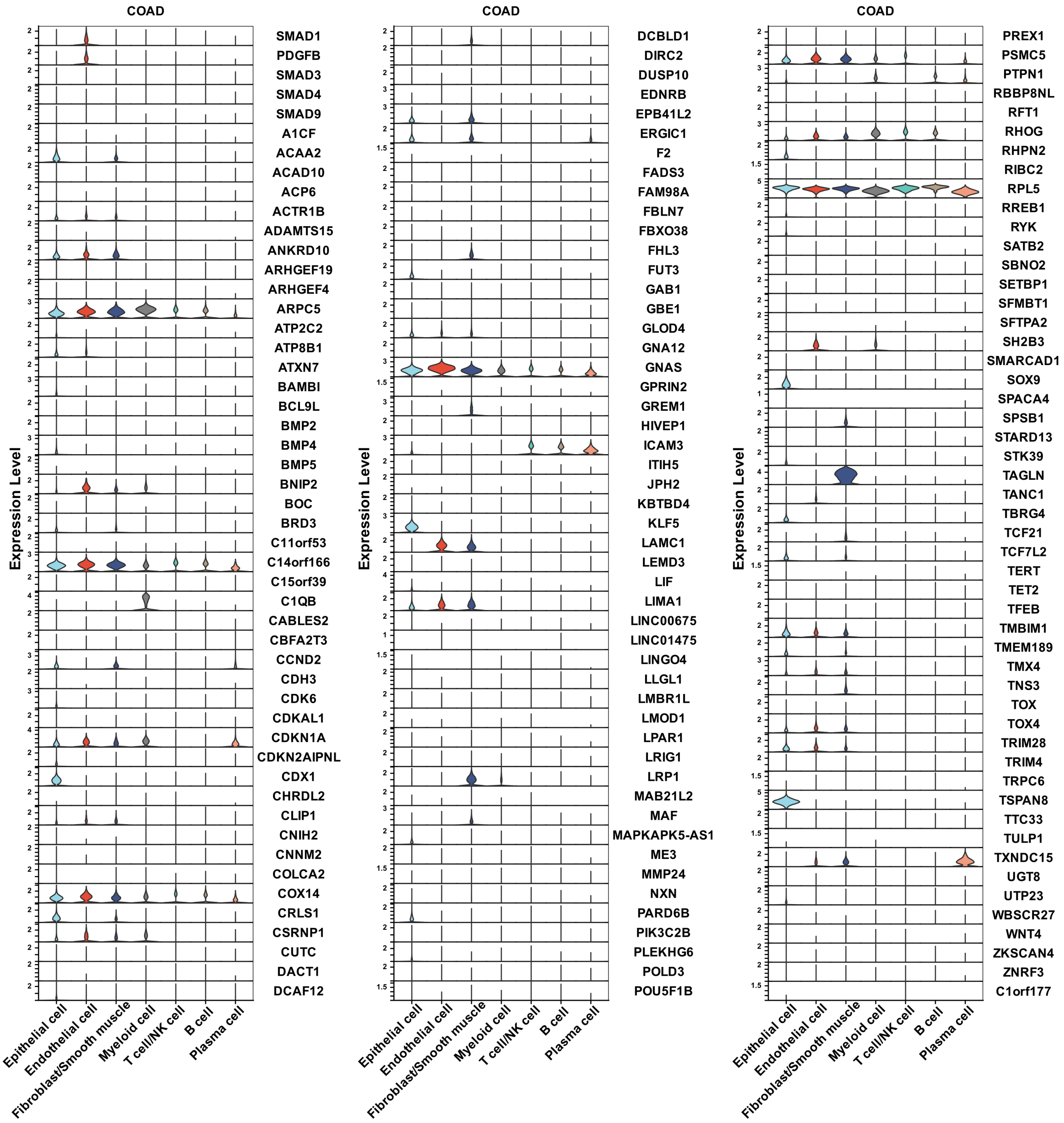
COAD
Expression Level
SMAD1
PDGFB
SMAD3
SMAD4
SMAD9
A1CF
ACAA2
ACAD10
ACP6
ACTR1B
ADAMTS15
ANKRD10
ARHGEF19
ARHGEF4
ARPC5
ATP2C2
ATP8B1
ATXN7
BAMBI
BCL9L
BMP2
BMP4
BMP5
BNIP2
BOC
BRD3
C11orf53
C14orf166
C15orf39
C1QB
CABLES2
CBFA2T3
CCND2
CDH3
CDK6
CDKAL1
CDKN1A
CDKN2AIPNL
CDX1
CHRDL2
CLIP1
CNIH2
CNNM2
COLCA2
COX14
CRLS1
CSRNP1
CUTC
DACT1
DCAF12
DCBLD1
DIRC2
DUSP10
EDNRB
EPB41L2
ERGIC1
F2
FADS3
FAM98A
FBLN7
FBXO38
FHL3
FUT3
GAB1
GBE1
GLOD4
GNA12
GNAS
GPRIN2
GREM1
HIVEP1
ICAM3
ITIH5
JPH2
KBTBD4
KLF5
LAMC1
LEMD3
LIF
LIMA1
LINC00675
LINC01475
LINGO4
LLGL1
LMBR1L
LMOD1
LPAR1
LRIG1
LRP1
MAB21L2
MAF
MAPKAPK5-AS1
ME3
MMP24
NXN
PARD6B
PIK3C2B
PLEKHG6
POLD3
POU5F1B
PREX1
PSMC5
PTPN1
RBBP8NL
RFT1
RHOG
RHPN2
RIBC2
RPL5
RREB1
RYK
SATB2
SBNO2
SETBP1
SFMBT1
SFTPA2
SH2B3
SMARCAD1
SOX9
SPACA4
SPSB1
STARD13
STK39
TAGLN
TANC1
TBRG4
TCF21
TCF7L2
TERT
TET2
TFEB
TMBIM1
TMEM189
TMX4
TNS3
TOX
TOX4
TRIM28
TRIM4
TRPC6
TSPAN8
TTC33
TULP1
TXNDC15
UGT8
UTP23
WBSCR27
WNT4
ZKSCAN4
ZNRF3
C1orf177
Epithelial cell
Endothelial cell
Fibroblast/Smooth muscle
Myeloid cell
T cell/NK cell
B cell
Plasma cell

B

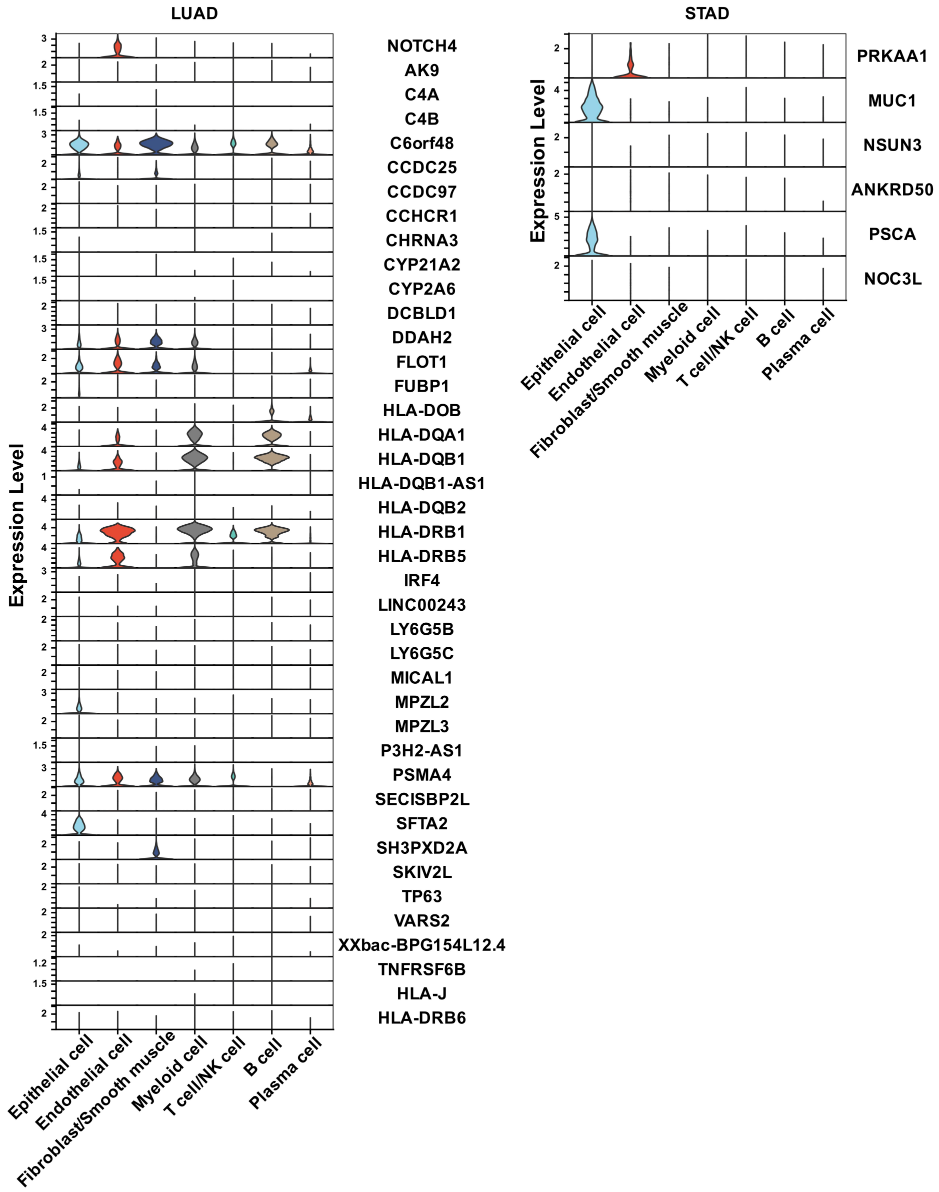

Figure S6. Cancer-associated genes were expressed in diverse cell types in tumor tissues. (A). Colon adenocarcinoma (COAD)-associated genes' expression pattern in colorectal tumor tissues. (**B).** Lung adenocarcinoma (LUAD) and Stomach adenocarcinoma (STAD)- associated genes' expression patterns in lung and gastric tumor tissues, respectively. Associated gene list was collected from published studies (see Methods). Expression level: normalized mRNA expression (see Methods).

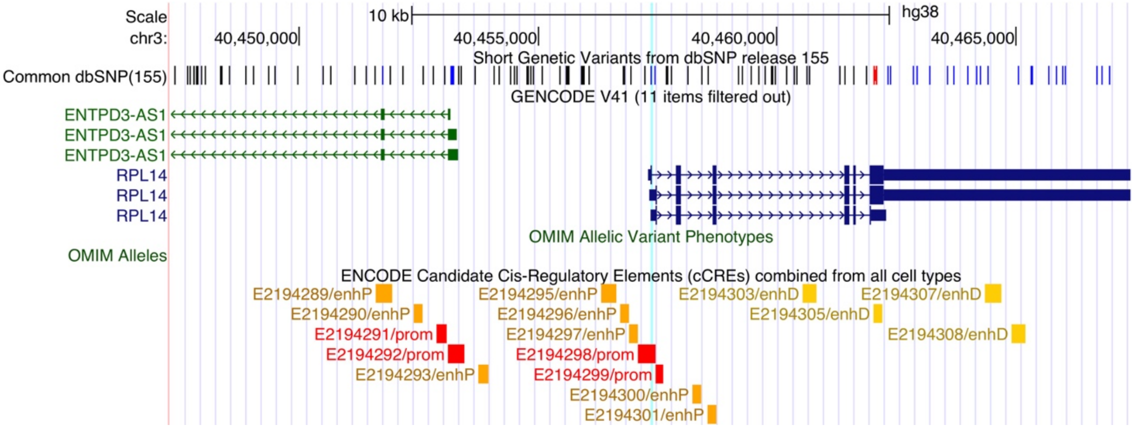

Figure S7. Genomic annotation of the NPC-associated SNP rs2276868. Light blue vertical line: The position of the target SNP rs2276868. The overlap between the light blue vertical line and a *cis*-regulatory element (E2194298, promoter) reported by the ENCODE consortium as well as the 5'UTR region of *RPL14* implicated that rs2276868 has the potential to regulate the gene expression of *RPL14* in *cis*. The figure was produced by UCSC genome browser (https://genome.ucsc.edu/).

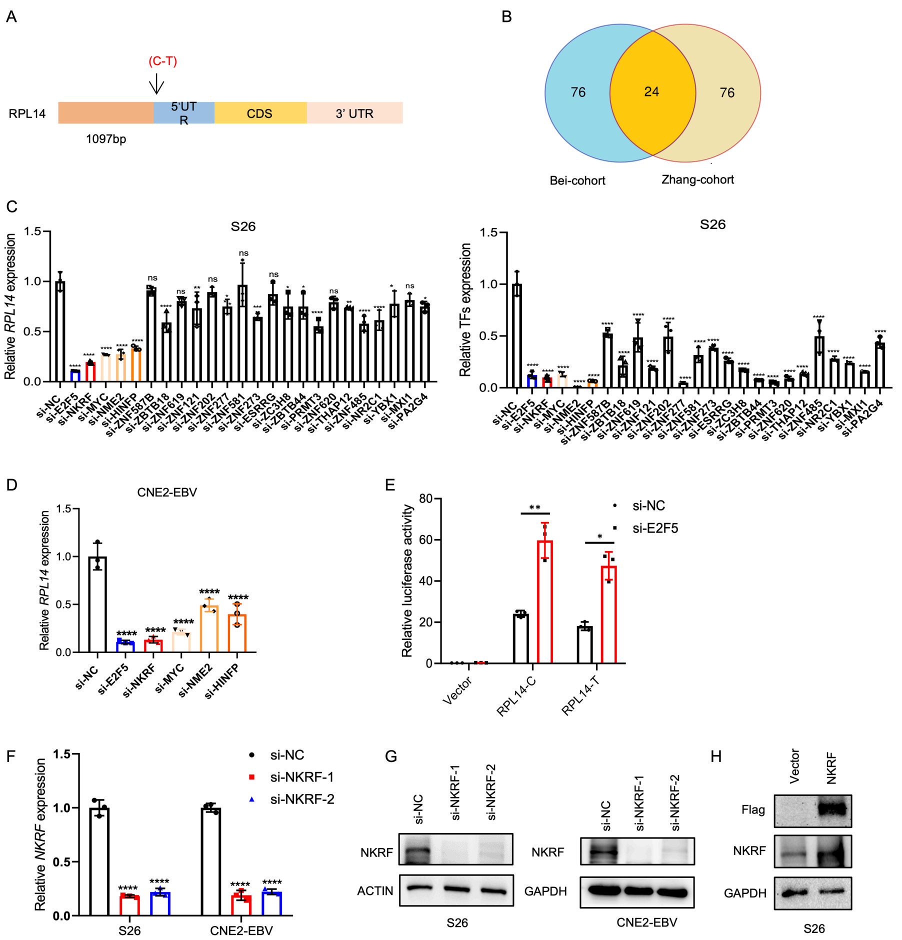

Figure S8. Candidate transcription factors of RPL14. (A). Schematic diagram presented the DNA fragments of RPL14. (**B**)**.** Venn diagram showed the overlap transcription factors correlated with RPL14 expression in two independent NPC cohorts. (**C**). S26 cells were transfected with indicated siRNAs targeting candidate transcription factors of RPL14 or control siRNA. RT-qPCR was performed to detect the mRNA expression of *RPL14* (left) or transcription factors (right). (**D**). CNE2-EBV cells were transfected with indicated siRNAs targeting candidate transcription factors of RPL14 or control siRNA. RT-qPCR was performed to detect the mRNA expression of *RPL14*. (**E**). Relative luciferase activity in 293T cells co-transfected with the rs2276868 -[C] or -[T] plasmids and E2F5 siRNA. (**F-G**). RT-qPCR (F) and western blotting (G) results showed the knockdown efficiency of NKRF in S26 and CNE2-EBV cells transfected with NKRF siRNAs or control siRNA. (**H**)**.** S26 cells were transfected with NKRF overexpression plasmids or control vector. Western blot was performed to evaluate the protein levels of NKRF. GAPDH or ACTIN was used as internal control. Between-group comparisons: t-test for two groups, one-way ANOVA followed by Sidak's post hoc test (comparisons among all groups) or Dunnett's post hoc test (comparisons with the control group) for ≥2 groups comparisons. *P<0.05, **P<0.01, ***P<0.001, ****P<0.0001.

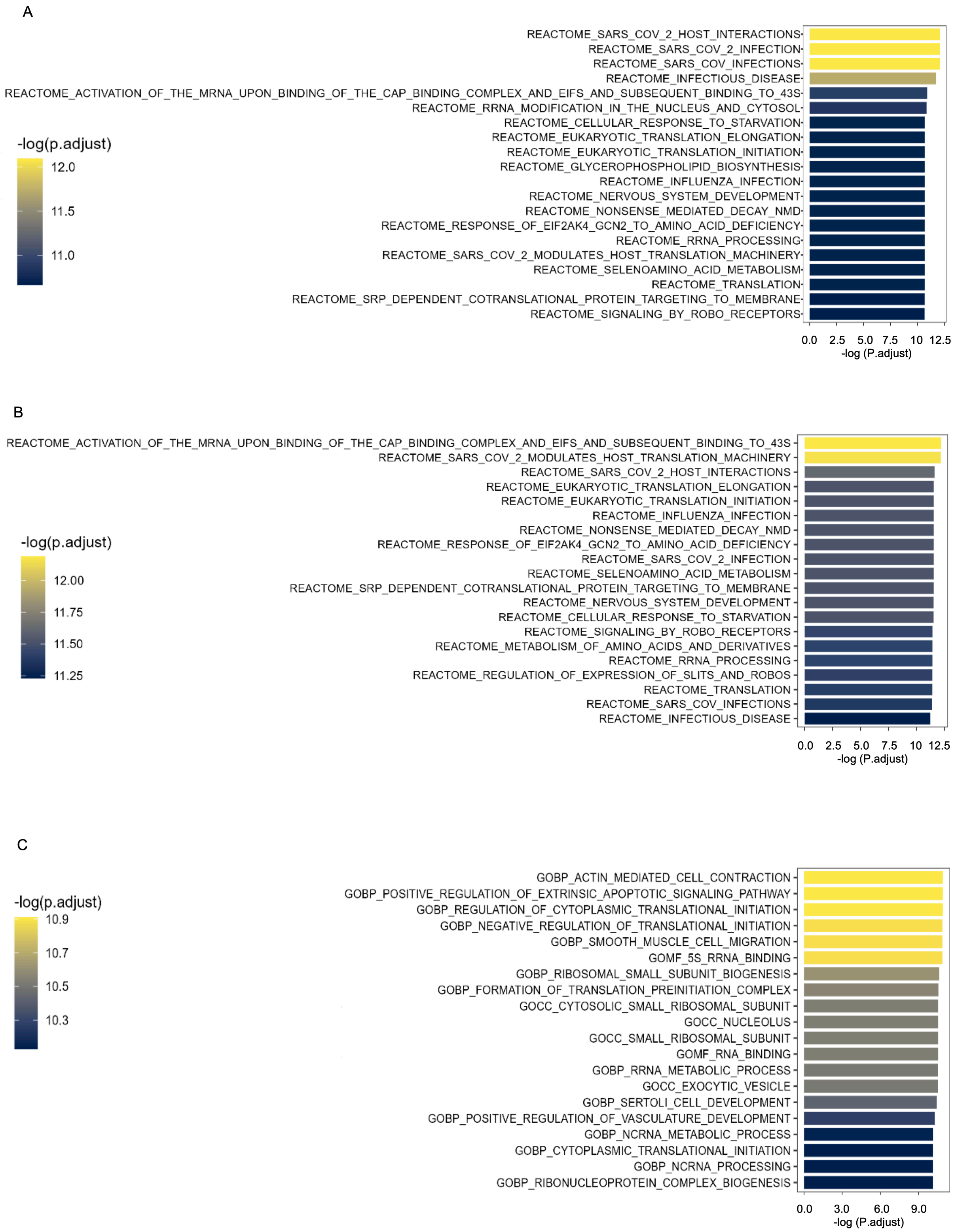
A
REACTOME_SARS_COV_2_HOST_INTERACTIONS
REACTOME_SARS_COV_2_INFECTION
REACTOME_SARS_COV_INFECTIONS
REACTOME_INFECTIOUS_DISEASE
REACTOME_ACTIVATION_OF_THE_MRNA_UPON_BINDING_OF_THE_CAP_BINDING_COMPLEX_AND_EIFS_AND_SUBSEQUENT_BINDING_TO_43S
REACTOME_RRNA_MODIFICATION_IN_THE_NUCLEUS_AND_CYTOSOL
REACTOME_CELLULAR_RESPONSE_TO_STARVATION
REACTOME_EUKARYOTIC_TRANSLATION_ELONGATION
REACTOME_EUKARYOTIC_TRANSLATION_INITIATION
REACTOME_GLYCEROPHOSPHOLIPID_BIOSYNTHESIS
REACTOME_INFLUENZA_INFECTION
REACTOME_NERVOUS_SYSTEM_DEVELOPMENT
REACTOME_NONSENSE_MEDIATED_DECAY_NMD
REACTOME_RESPONSE_OF_EIF2AK4_GCN2_TO_AMINO_ACID_DEFICIENCY
REACTOME_RRNA_PROCESSING
REACTOME_SARS_COV_2_MODULATES_HOST_TRANSLATION_MACHINERY
REACTOME_SELENOAMINO_ACID_METABOLISM
REACTOME_TRANSLATION
REACTOME_SRP_DEPENDENT_COTRANSLATIONAL_PROTEIN_TARGETING_TO_MEMBRANE
REACTOME_SIGNALING_BY_ROBO_RECEPTORS
-log(p.adjust)
12.0
11.5
11.0
0.0 2.5 5.0 7.5 10 12.5
-log (P.adjust)
B
REACTOME_ACTIVATION_OF_THE_MRNA_UPON_BINDING_OF_THE_CAP_BINDING_COMPLEX_AND_EIFS_AND_SUBSEQUENT_BINDING_TO_43S
REACTOME_SARS_COV_2_MODULATES_HOST_TRANSLATION_MACHINERY
REACTOME_SARS_COV_2_HOST_INTERACTIONS
REACTOME_EUKARYOTIC_TRANSLATION_ELONGATION
REACTOME_EUKARYOTIC_TRANSLATION_INITIATION
REACTOME_INFLUENZA_INFECTION
REACTOME_NONSENSE_MEDIATED_DECAY_NMD
REACTOME_RESPONSE_OF_EIF2AK4_GCN2_TO_AMINO_ACID_DEFICIENCY
REACTOME_SARS_COV_2_INFECTION
REACTOME_SELENOAMINO_ACID_METABOLISM
REACTOME_SRP_DEPENDENT_COTRANSLATIONAL_PROTEIN_TARGETING_TO_MEMBRANE
REACTOME_NERVOUS_SYSTEM_DEVELOPMENT
REACTOME_CELLULAR_RESPONSE_TO_STARVATION
REACTOME_SIGNALING_BY_ROBO_RECEPTORS
REACTOME_METABOLISM_OF_AMINO_ACIDS_AND_DERIVATIVES
REACTOME_RRNA_PROCESSING
REACTOME_REGULATION_OF_EXPRESSION_OF_SLITS_AND_ROBOS
REACTOME_TRANSLATION
REACTOME_SARS_COV_INFECTIONS
REACTOME_INFECTIOUS_DISEASE
-log(p.adjust)
12.00
11.75
11.50
11.25
0.0 2.5 5.0 7.5 10 12.5
-log (P.adjust)
C
GOBP_ACTIN_MEDIATED_CELL_CONTRACTION
GOBP_POSITIVE_REGULATION_OF_EXTRINSIC_APOPTOTIC_SIGNALING_PATHWAY
GOBP_REGULATION_OF_CYTOPLASMIC_TRANSLATIONAL_INITIATION
GOBP_NEGATIVE_REGULATION_OF_TRANSLATIONAL_INITIATION
GOBP_SMOOTH_MUSCLE_CELL_MIGRATION
GOMF_5S_RRNA_BINDING
GOBP_RIBOSOMAL_SMALL_SUBUNIT_BIOGENESIS
GOBP_FORMATION_OF_TRANSLATION_PREINITIATION_COMPLEX
GOCC_CYTOSOLIC_SMALL_RIBOSOMAL_SUBUNIT
GOCC_NUCLEOLUS
GOCC_SMALL_RIBOSOMAL_SUBUNIT
GOMF_RNA_BINDING
GOBP_RRNA_METABOLIC_PROCESS
GOCC_EXOCYTIC_VESICLE
GOBP_SERTOLI_CELL_DEVELOPMENT
GOBP_POSITIVE_REGULATION_OF_VASCULATURE_DEVELOPMENT
GOBP_NCRNA_METABOLIC_PROCESS
GOBP_CYTOPLASMIC_TRANSLATIONAL_INITIATION
GOBP_NCRNA_PROCESSING
GOBP_RIBONUCLEOPROTEIN_COMPLEX_BIOGENESIS
-log(p.adjust)
10.9
10.7
10.5
10.3
0.0 3.0 6.0 9.0
-log (P.adjust)

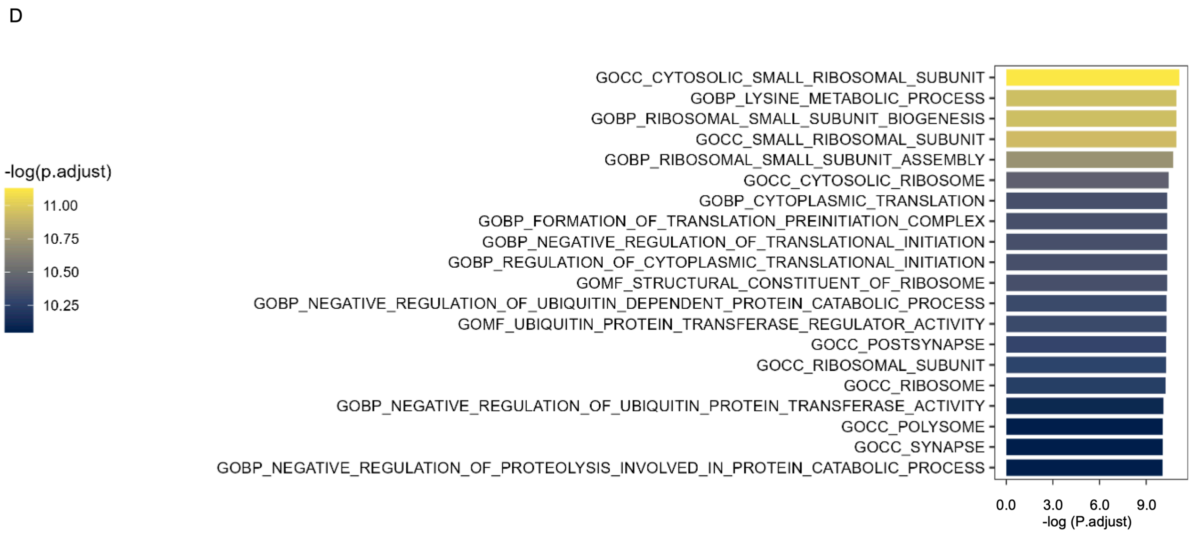

Figure S9. The top 20 pathways significantly correlated with the expression of *RPL14* in tumor tissues (Bulk RNA seq data). (**A).** REACTOME pathways results from the Bei-lab dataset (n_{sample}=93). (**B).** REACTOME pathways results from the Zhang-lab dataset (n_{sample}=113). (**C**). GO ontology pathways results from the Bei-lab dataset (nsample=93). (**D**). GO ontology pathways results from the Zhang-lab dataset (n_{sample}=113).

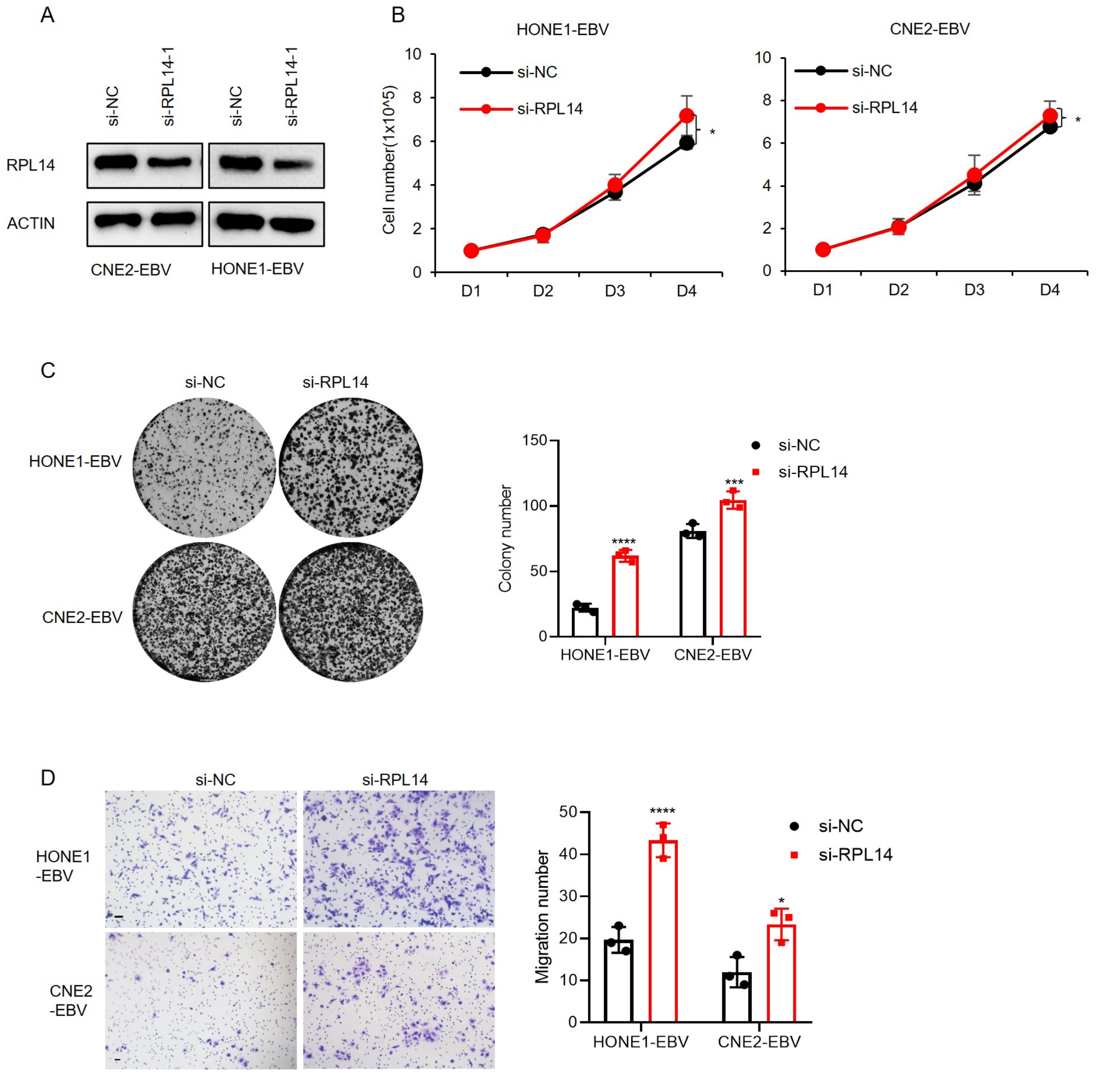

Figure S10. Knockdown of RPL14 promoted cell proliferation and migration of NPC cells. (A). HONE1-EBV and CNE2-EBV cells were transfected siRNAs targeting RPL14 or control siRNA. Western blotting results demonstrated the knockdown efficiency. (**B)**. Cell growth curves were measured with cells described in A. (**C). ** Colony formation assays were performed with cells described in A. The statistical data were presented at the right. (**D).** Transwell assays were performed with cell described in A and the corresponding statistical analysis were demonstrated at the right. Scale bar, 100 μm. Statistical methods for between-group comparisons: t test. **P*<0.05, **P<0.01, ****P*<0.001, *****P*<0.0001.

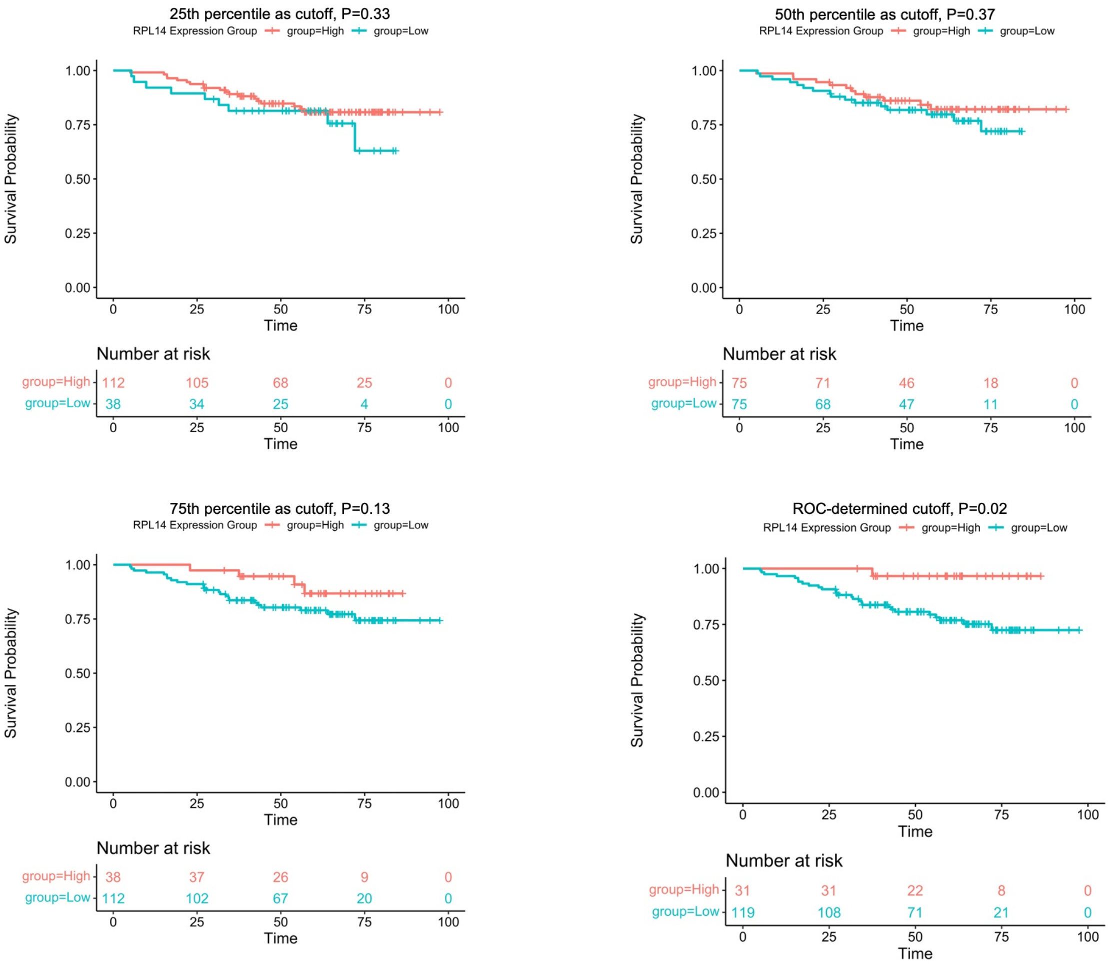

Figure S11. Sensitivity Kaplan-Meier analyses for the association between *RPL14* expression level and overall survival (OS) of 150 NPC patients in Chen et al. cohort. *RPL14* expression levels were normalized using *EPCAM* expression to account for variations in the proportion of epithelial cells in tumor tissues, and subsequently scaled to a mean of 0 and a variance of 1. Survival curves represent high (red) or low (blue) expression of *RPL14*, determined using different cutoff quartiles or receiver operating characteristic as indicated. P: P value from log-rank test. ROC: Receiver-operating characteristic curve.

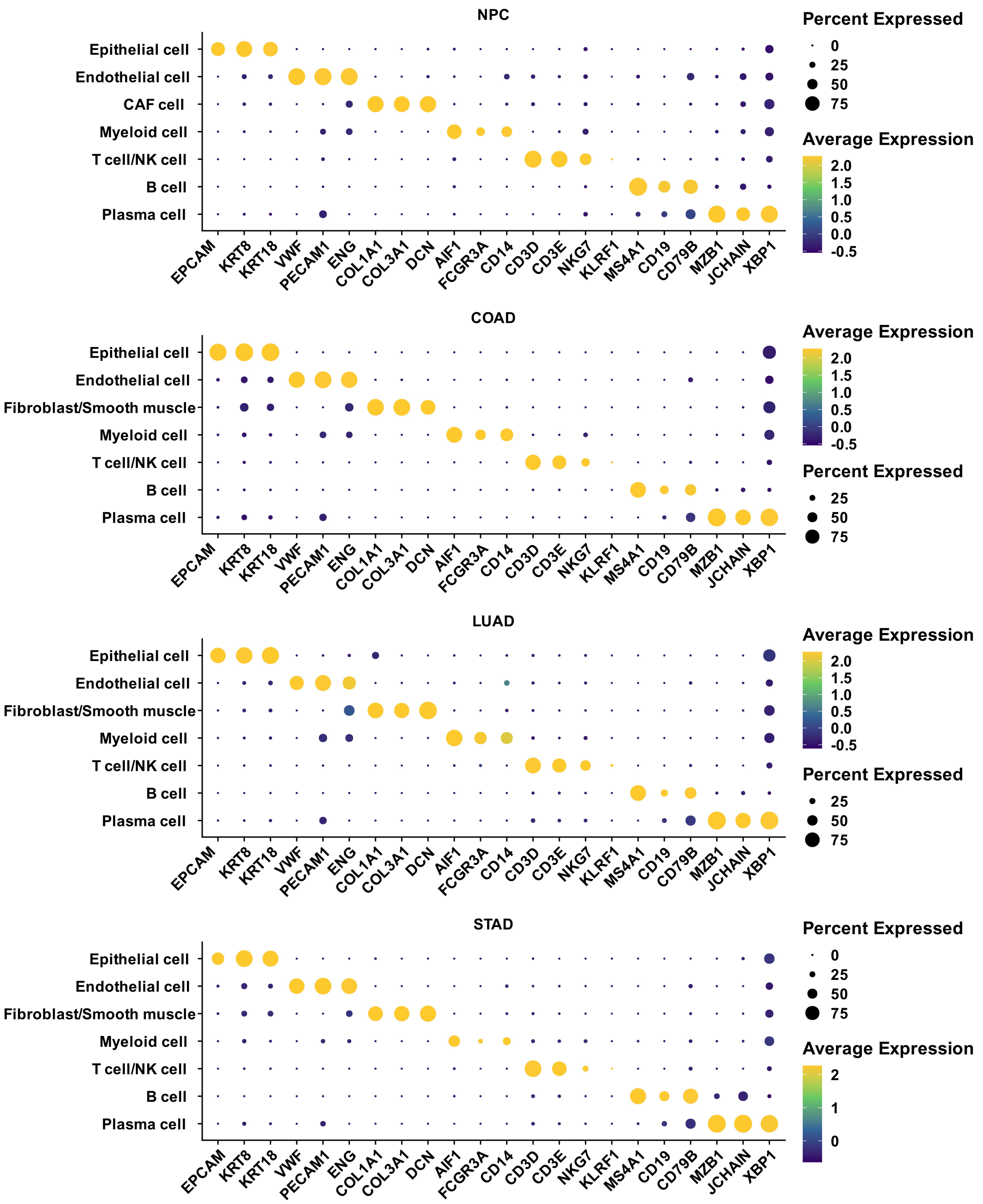

Figure S12. Normalized expression of marker genes in identified cell types using scRNA-seq data for tumor tissues. NPC: nasopharyngeal carcinoma, COAD: colon adenocarcinoma, LUAD: Lung adenocarcinoma, STAD: stomach adenocarcinoma. The genes listed were specific marker genes for the major cell types listed in Y axis. Percent expressed: percentage of cells that express a certain marker gene among cells annotated to a given cell type. Average expression: the mean of the normalized expression level of a given marker gene among cells annotated to a given cell type.
