# Supplementary figures and images for: Whole-exome sequencing association study reveals genetic effects on tumor microenvironment components in nasopharyngeal carcinoma
Source: J Clin Invest. 2025 Jan 2;135(1):e182768. doi: 10.1172/JCI182768 (PMC11684818; doi:10.1172/JCI182768)

Fig.5I

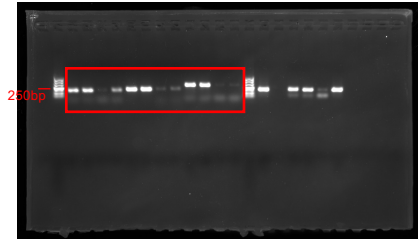

Fig.6D

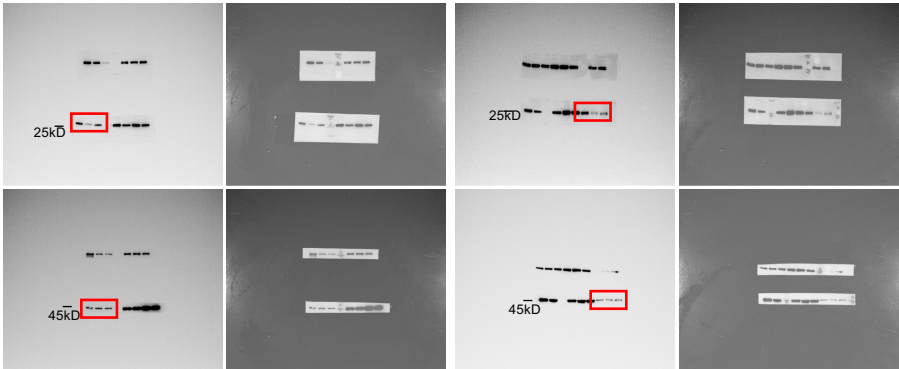

Fig.6F

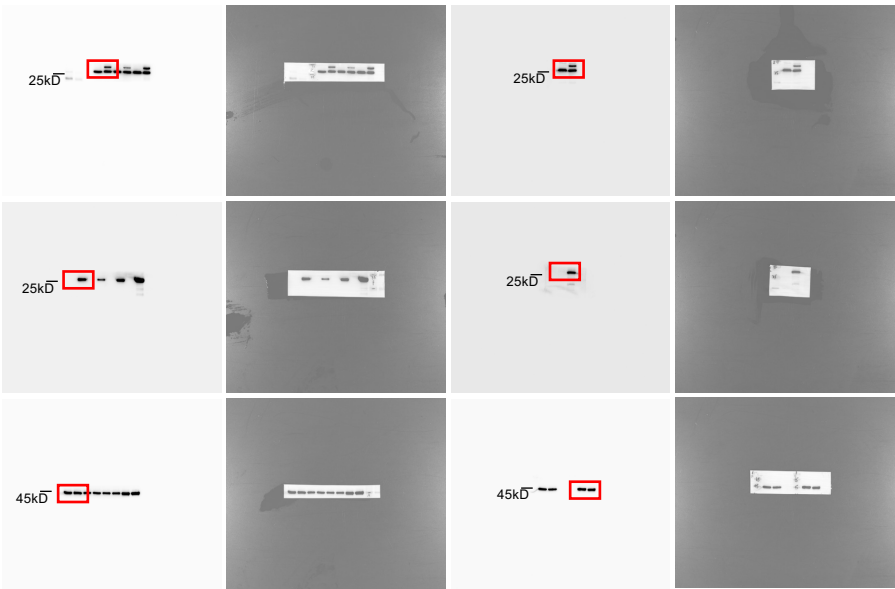

Fig.7A

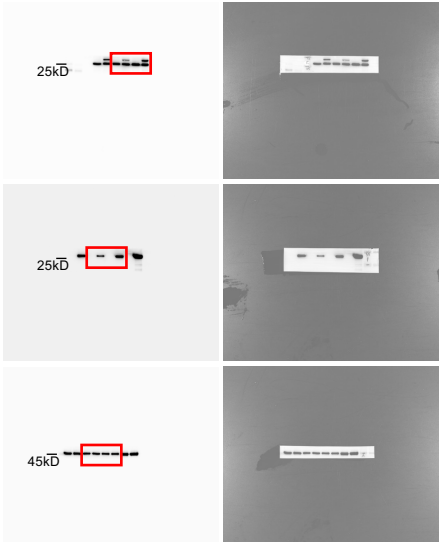

Fig.8B

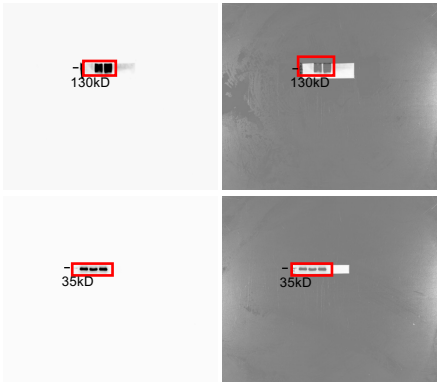

Fig.S8G

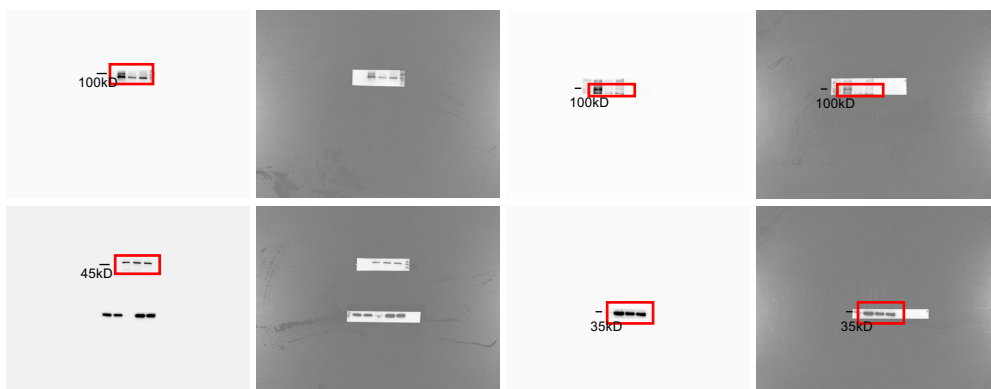

Fig.S8H

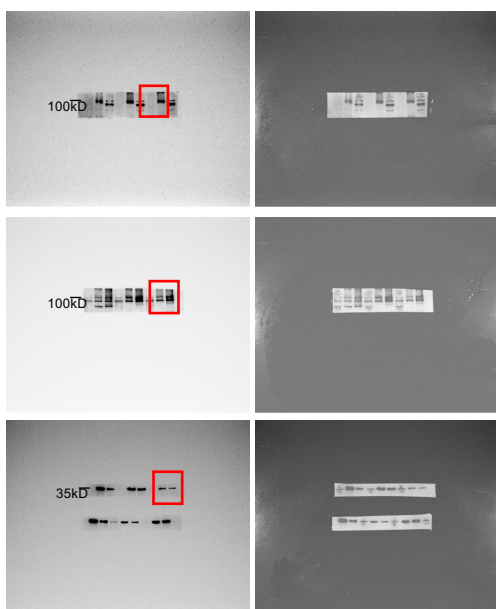

Fig.S10A

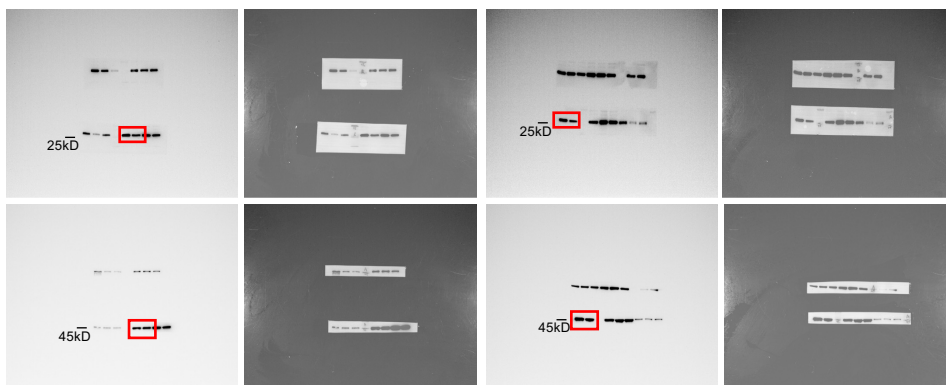

Supplement: Unedited blot and gel images [file jci-135-182768-s145.pdf]
